# Phase Change Mechanism and Safety Control During the Shutdown and Restart Process of Supercritical Carbon Dioxide Pipelines

**DOI:** 10.3390/molecules31010104

**Published:** 2025-12-26

**Authors:** Xinze Li, Dezhong Wang, Weijie Zou, Jianye Li, Xiaokai Xing

**Affiliations:** 1 School of Engineering, China University of Petroleum (Beijing) at Karamay, Karamay 834000, China; wangdezhong@st.cupk.edu.cn (D.W.); 2023216899@st.cupk.edu.cn (J.L.); xingxiaokai@vip.sina.com (X.X.); 2 Xinjiang Key Laboratory of Multi-Medium Pipeline Safety Transportation, Urumqi 830011, China; 3 PipeChina West Pipeline Co., Ltd., Urumqi 830011, China; zwj17340465658@163.com

**Keywords:** carbon dioxide pipeline, CO_2_ phase change characteristics, shutdown and restart, phase migration path, safety control

## Abstract

Supercritical CO_2_ pipeline transportation is a crucial link in Carbon Capture, Utilization, and Storage (CCUS). Compared with traditional oil and gas pipelines, if a supercritical CO_2_ pipeline is shut down for an excessively long time, the phase state of CO_2_ may transform into a gas–liquid two-phase state. It is urgently necessary to conduct research on the phase change mechanism and safety control during the restart process of gas–liquid two-phase CO_2_ pipelines. Based on a certain planned supercritical carbon dioxide pipeline demonstration project, this paper proposes a new pipeline safety restart scheme that actively seeks the liquefaction of gaseous CO_2_ inside the pipeline by injecting liquid-phase CO_2_ at the initial station. Through numerical simulation and experimental methods, the co-variation laws of parameters such as temperature, pressure, density, and phase state during the pipeline restart process were revealed. It was found that the pipeline shutdown and restart process could be subdivided into four stages: shutdown stage, liquefaction stage, pressurization stage, and displacement stage. The phase transition line would form a closed curve that is approximately trapezoidal. It is suggested to optimize the restart scheme from aspects such as reducing the restart time, controlling the pressure rise rate, and saving CO_2_ consumption. It is proposed that the liquid holdup of CO_2_ fluid in the pipe at the initial moment of restart and the mass flow rate of CO_2_ injected at the initial station during the restart process are the main controlling factors affecting the evolution of the phase path of pipeline restart. For the demonstration project, the specific critical threshold values are given. The research results can provide a certain theoretical guidance and reference basis for the safe restart method of supercritical CO_2_ pipelines.

## 1. Introduction

Carbon Capture, Utilization, and Storage (CCUS) is internationally recognized as one of the three major carbon reduction pathways and plays an irreplaceable and important role in the process of carbon neutrality. CO_2_ transportation is a key link in CCUS technology, and pipeline transportation is crucial for realizing large-scale and long-distance CO_2_ delivery [1,2,3]. Restricted by geographical distribution, CO_2_ emission sources and utilization/storage hubs require spatial transfer. Considering the safety, stability, and economic feasibility of existing transportation methods, pipeline transportation is the optimal way to achieve large-scale CO_2_ transportation, making the construction of long-distance CO_2_ pipeline infrastructure imperative [4,5,6].

Globally, over 50 years of CO_2_ pipeline engineering practice has been accumulated. More than 50 independent CO_2_ pipelines are currently in operation, with a total network length exceeding 1.0 × 10^4^ km, most of which are located in the United States. The total annual pipeline transportation capacity has reached 86 million tons, and nearly 80% of the existing pipelines adopt supercritical transportation technology. The designed pipeline pressure ranges from 10 MPa to 20 MPa. The maximum annual transportation capacity of a single pipeline is 19.3 million tons, and the maximum designed pipe diameter is DN750 [7,8]. For developing countries, CO_2_ pipeline construction started late on a small scale, and related technologies and supporting policies are relatively backward. In July 2023, China’s first onshore CO_2_ pipeline with an annual transportation capacity exceeding one million tons—connecting Sinopec Qilu Petrochemical to Shengli Oilfield—was successfully completed and put into operation. With a main line length of 109 km, it marks China’s first realization of long-distance and high-pressure dense-phase CO_2_ pipeline transportation.

Due to significant differences in phase characteristics and transportation properties between CO_2_ and crude oil or natural gas, the processes and operation technologies of crude oil and natural gas pipelines cannot be directly applied to CO_2_ pipelines. For example, there are no safe operation and maintenance procedures for transient processes such as shutdown and restart, venting, water hammer, and leakage emergency rescue during CO_2_ pipeline operation, which fails to meet the requirements for efficient transportation and operational safety guarantee throughout the whole life cycle of supercritical CO_2_ pipelines. To address the great challenges in engineering practice, it is necessary to strengthen the difference analysis between CO_2_ pipelines and traditional crude oil/natural gas pipelines, identify key focus areas, clarify the underlying mechanisms, and develop core technologies.

During the normal operation or abnormal conditions of supercritical CO_2_ pipelines, transient transportation processes such as shutdown and restart may also occur. In the event of a leakage accident, an emergency shutdown should be implemented, followed by a restart after the pipeline system is repaired. In addition, shutdown and restart operations are performed during routine pipeline maintenance. For crude oil pipeline shutdown and restart, the decrease in temperature after shutdown leads to increased crude oil viscosity, wax crystal precipitation, and thickened wax deposition, which may form a network structure across the entire pipeline cross-section. The pressure required for pipeline restart may exceed the designed pressure, posing an overpressure risk to the pipeline. For natural gas pipeline shutdown and restart, hydrate formation may occur during shutdown, and there is a risk of pipeline blockage during restart.

Regarding the shutdown and restart issue of supercritical CO_2_ pipelines, there is a temperature difference between the fluid inside the pipeline and the surrounding soil. During the shutdown process, the pressure of CO_2_ decreases with the drop in temperature, making the fluid prone to phase change. Physical parameters such as fluid density and specific heat capacity undergo stepwise changes, leading to severe fluctuations in the mass of CO_2_ fluid inside the pipeline. This will cause a pulsating impact on the pipeline and endanger its safety. The final state of pipeline shutdown serves as the initial boundary for restart, and whether phase change occurs in the fluid inside the pipeline is directly related to the safety of pipeline restart. Meanwhile, gaseous CO_2_ may affect the plunger pumps used for oilfield formation injection at the pipeline terminal, endangering the safe operation of equipment.

At present, there is a lack of systematic research on the restart process of supercritical CO_2_ pipelines. GB/T 42797-2023 [9] “Pipeline Transportation Systems for Carbon Dioxide Capture, Transportation, and Geological Storage” only points out that, during the shutdown period of supercritical CO_2_ pipelines, the pipeline pressure should be maintained to prevent phase change and free water generation, and the temperature drop should also be controlled within the range that avoids the occurrence of two-phase flow. It can be seen that, from the perspective of engineering safety, the standard requires that the restart operation of CO_2_ pipelines should be completed before the fluid vaporizes.

To focus on solving the problem of pipeline restart after the fluid phase state changes due to an excessively long pipeline shutdown time, this study was conducted using a combination of numerical simulation and experimental methods. The innovation of this paper is mainly reflected in three aspects. First, it is the first time to propose a new idea of injecting liquid-phase CO_2_ at the initial station to make the gas–liquid two-phase CO_2_ inside the pipeline first liquefy, then pressurize, and finally displace. Second, through the combination of numerical simulation and experimental verification, it is the first time to clarify the four stages of the shutdown and restart process of supercritical CO_2_ pipelines (shutdown stage, liquefaction stage, pressurization stage, and displacement stage), reveal the evolution laws of parameters such as temperature, pressure, density, and liquid holdup during the process, and present the trapezoidal closed path of phase migration throughout the process, thereby clarifying the phase evolution mechanism. Third, to determine the safe restart control method of the pipeline, by evaluating key parameters such as liquefaction time, pressure rise time, pressure rise rate, and CO_2_ consumption during the restart process, the critical threshold values of the main controlling factors, such as allowable liquid holdup of the pipeline, injection mass flow rate, and ambient temperature, are given, providing directly applicable quantitative standards for the safety control of the restart process.

From the perspective of practical application value, the number of long-distance supercritical CO_2_ pipeline projects is increasing. However, the existing CO_2_ pipeline transportation standards do not specify the safe restart control measures after pipeline shutdown, which brings great challenges to the safe operation of pipelines. The technical scheme proposed in this study can be directly applied to under-construction and planned CCUS pipeline projects, ensuring that the pipeline can be restarted under safe and controllable conditions, while reducing CO_2_ injection loss and improving engineering economy. It has important engineering practical significance for promoting the large-scale implementation of CCUS technology.

## 2. Research Status

### 2.1. Transient Hydraulic and Thermal Model of Supercritical CO_2_ Pipelines

Currently, the number of research achievements on the transient characteristics of supercritical CO_2_ pipelines is relatively limited. To better understand the variation characteristics of fluids inside the pipeline during the rupture, leakage, and venting of CO_2_ pipelines, many scholars have studied the dynamic change process of fluids in CO_2_ pipelines after rupture by establishing transient models. Mahgerefteh et al. [10] numerically solved the fluid conservation equations using the method of characteristics, realizing the simulation analysis of transient flow during leakage. Subsequently, a comparative analysis was conducted on the changes in physical parameters when CO_2_ pipelines and natural gas pipelines rupture under the same conditions. The results showed that the entire pressure relief process of the two types of pipelines is not significantly different. However, the numerical solution of the gas discharge rate in CO_2_ pipelines is significantly higher than that in natural gas pipelines. Munkejord et al. [11] studied the pressure relief process of CO_2_ mixtures containing methane impurities using the SRK equation and drift-flux model. Browna et al. [12] proposed a two-fluid transient flow model. By comparing and analyzing the predicted transient pressure and temperature curves of the pipeline with the prediction results based on the simplified homogeneous equilibrium model (HEM), the error was found to be small. Meanwhile, the model simulated the leakage process of CO_2_ pipelines under high-pressure conditions. Sergey Martynova et al. [13] established a compressible computational fluid dynamics (CFD) model to predict the formation of dry ice during the transient decompression of CO_2_ pipelines. Based on the homogeneous equilibrium mixture (HEM) assumption, this model uses the extended Peng–Robinson equation of state to predict the physical properties of CO_2_ in gaseous, liquid, and solid states. The results showed that the model has better predictive ability for the physical properties of CO_2_ under various physical states. The research team led by Munkejorda [14] developed a new combined model for CO_2_ pipelines. By integrating thermodynamic principles and flow dynamic characteristics, this model can accurately simulate the flow states of gas–liquid two-phase media containing multiple components under different working conditions, including changes during normal operation and emergency states. Liu et al. [15] established a multiphase flow dynamics model for simulating the decompression process of high-pressure CO_2_ pipelines and found that non-equilibrium phase transition has a significant impact on the decompression wave velocity. Lu et al. [16] constructed a one-dimensional flow model of CO_2_ along the pipeline, which can couple hydraulic and thermal factors during the flow process, enabling hydraulic calculations along the pipeline during slow transient processes. Fang et al. [17] established a dynamic simulation model for two-phase flow in CO_2_ pipelines containing impurities. Zhao et al. [18] used MATLAB and C++ programming technologies, combined with the phase characteristics of CO_2_, to establish a one-dimensional transient simulation calculation model for flow fluctuations in supercritical/dense-phase CO_2_ pipelines containing impurities and conduct numerical solutions for this model.

Although existing studies have made certain progress in transient models such as venting, leakage, and flow fluctuations of supercritical CO_2_ pipelines, research on hydraulic and thermal calculations involving the transient process of pipeline shutdown and restart is scarce. It is urgent to study the hydraulic and thermal calculation models required to accurately describe the entire process of pipeline shutdown and restart through experiments.

### 2.2. In-Pipe Flow Parameters and Phase Changes During the Shutdown and Restart Process of Supercritical CO_2_ Pipelines

In the actual operation of long-distance supercritical CO_2_ transportation pipelines, the pipeline system will inevitably experience various dynamic operating conditions, including unsteady conditions such as planned and sudden shutdowns, restart-after-shutdown, leakage, and venting. Compared with stable operating conditions, these transient conditions are more dangerous and cause greater harm. Once the above transient conditions occur, the operating parameters such as throughput, pressure, and temperature of the CO_2_ pipeline will suddenly change drastically, and the CO_2_ fluid in the pipeline will correspondingly form transient flow. The CO_2_ phase point, determined by the pressure and temperature under transient conditions, may drop to near the bubble point line below the gas–liquid two-phase region, causing vaporization of the CO_2_ fluid in the pipeline. This may exert strong impacts on the pipeline and related auxiliary equipment, leading to damage to the pipeline and equipment, and seriously endangering the safe operation of the pipeline.

At present, research on the changes in the in-pipe parameters during the shutdown and restart process of supercritical CO_2_ pipelines mainly focuses on the shutdown process, with relatively few studies on the restart process. Wang conducted a transient simulation on a 160 km-long CO_2_ transportation pipeline, simulating the shutdown and restart conditions of the pipeline. Liu constructed a simulation model based on the OLGA commercial software (v.2020.1.0) for the shutdown and restart conditions of supercritical CO_2_ pipelines, carried out dynamic simulation research combined with CO_2_ phase change characteristics, revealed the variation laws of parameters such as pressure wave propagation and temperature gradient during the pipeline shutdown and restart process, and analyzed the influence laws of key operating parameters such as injection mass flow rate and initial operating pressure on the safe shutdown time. Chen et al. [19,20] used OLGA to simulate the changes in operating parameters during the shutdown and restart process of CO_2_ pipelines, combined with the physical properties and phase diagram analysis of CO_2_ containing impurities, provided the process parameters such as temperature and flow rate at the initial shutdown of CO_2_ pipelines, and predicted the hydrate formation position. Zhao Qing [18] constructed a numerical model for the pipeline shutdown and restart process based on the HYSYS commercial software (v.14.0) combined with the quasi-critical state characteristics of CO_2_, analyzed the dynamic parameter characteristics such as phase transition and pressure fluctuation of supercritical CO_2_ fluid during pipeline shutdown and restart, and revealed the influence of physical parameters near the critical point on the operational safety of CO_2_ pipelines.

Li’s research team conducted a systematic study on the synergistic variation laws of temperature, pressure, and phase state during the shutdown process of supercritical CO_2_ pipelines for a planned demonstration project in China, combining OLGA simulations, high-pressure sapphire kettle experiments, and other methods. It was found that, after the pipeline is shut down, the pressure and temperature along the line continuously decrease and tend to be consistent between the upstream and downstream after 3 h. The phase transition of the fluid into a gas–liquid two-phase state first occurs at the starting point and then continuously extends toward the terminal. There is a synergistic variation relationship between temperature and pressure, and the fluid phase transition path is supercritical phase ⟶ dense phase ⟶ liquid phase ⟶ gas–liquid equilibrium line (gas–liquid coexistence region). The temperature drop rate of the fluid in each phase state is 2.6–3.4 times the pressure drop rate. A scientific definition of the safe shutdown time of the pipeline is proposed, which refers to the time from the start of pipeline shutdown to the moment when the fluid at any position in the pipeline is about to enter the gas–liquid coexistence region [21].

However, after clarifying the variation laws of temperature, pressure, and phase state of the fluid inside the pipeline during the shutdown process, the industry is more concerned about the changes in temperature, pressure, and phase state of the fluid inside the pipeline during the restart process. If the pipeline shuts down due to reasons such as compressor failure, power outage, or pipeline leakage, and the fault cannot be eliminated in a timely manner within a certain period, the fluid inside the pipeline will inevitably vaporize when the shutdown time is excessively long. Especially in winter, the temperature difference between the fluid inside the pipeline and the surrounding soil is significant. Under the same shutdown pressure and temperature conditions, the fluid vaporization time will be earlier compared to that in summer. Once a gas–liquid two-phase state occurs in the pipeline fluid, how to successfully restart the pipeline and restore its operating state before shutdown becomes a challenge. During the restart process of the pipeline, the newly injected fluid at the initial station extrudes the fluid that has been shut down for a period of time, resulting in variations in pressure, temperature, and phase state of the fluid inside the pipeline. Accurately revealing the variation laws of these parameters is the key to formulating a safe pipeline restart plan.

### 2.3. Safety Control Measures for the Shutdown and Restart Process of Supercritical CO_2_ Pipelines

Currently, some scholars have initially proposed safety control measures based on the variation laws of parameters during the shutdown process of supercritical CO_2_ pipelines.

The Li research team conducted an analysis of the sensitive factors affecting pipeline shutdown time and concluded that reducing the outlet temperature of the initial station, increasing the pipeline operating pressure (process parameters), a higher soil ambient temperature, and a lower total pipeline heat transfer coefficient (environmental parameters) are conducive to extending the safe shutdown time of supercritical CO_2_ pipelines. Combined with a dimensional analysis, Python (v.3.12.4) was used for fitting to obtain a highly nonlinear regression calculation formula that maps seven variable parameters (independent variables) to the safe shutdown time of supercritical CO_2_ pipelines (dependent variable). Through a comparative analysis of the characteristics of in-pipe parameter fluctuations, temperature–pressure synergistic relationships, phase transition paths, and laws during shutdown under different seasons and boundary conditions, the safe shutdown process boundary ranges and functional expressions for the demonstration project in summer and winter were proposed, respectively [22,23].

Zhao et al. [24] suggested that pressure relief valves should be installed downstream of the pipeline, and part of the pressure relief process should be activated during shutdown. Key protection and monitoring of parameter changes during pipeline shutdown should be implemented to ensure the timely initiation of safe pressure relief operations and guarantee the safe operation of pipe sections. The standard DNV GL-RP-F104 [25] “Design and Operation of Carbon Dioxide Pipelines” points out that, during planned shutdowns, the fluid pressure inside the pipeline should be maintained at a high level to prevent CO_2_ vaporization [26]. When the pipeline is in a low ambient temperature environment, the rate of decrease in fluid temperature inside the pipeline should be considered to avoid the generation of two-phase flow. In low-temperature environments, the fluid temperature drop rate needs to be controlled to prevent two-phase flow. In high-temperature environments, an overpressure protection system should be installed to respond to pressure rises [27].

However, although existing studies have initially revealed the potential risks of excessively long shutdown times of supercritical CO_2_ pipelines, specific safe restart measures are rarely reported. For phase changes caused by different shutdown times (such as dense phase, gas–liquid two-phase, etc.), there is a lack of specific and quantitative restart condition judgment, restart methods (such as direct start-up, whether displacement is required, etc.), and corresponding risk control measures. The specific difficulties are as follows: (1) lack of safe restart schemes after CO_2_ vaporization and phase transition in the pipeline; (2) the corresponding relationship between liquid holdup (or vaporization rate) and restart risk is not clear, that is, whether the degree of vaporization is directly related to the restart risk level; (3) the adjustable variables during the restart process are limited to the phase state, mass flow rate, temperature, etc., of the newly injected fluid at initial station, and the optimization scheme of different parameter combinations remains to be studied; and (4) the restart boundaries such as pressure, temperature, phase state, and liquid holdup corresponding to different pipeline shutdown times are different, which increases the difficulty in formulating a unified safe restart scheme.

## 3. Establishment and Validity Verification of Pipeline Restart Model

### 3.1. Project Case

A demonstration project of a million-ton supercritical CO_2_ pipeline was selected as the object of analysis and research. The total length of this pipeline is 52 km, with an outer diameter of 273.1 mm, a wall thickness of 8 mm, an absolute roughness of the inner wall of 30 μm, a design pressure of 13 MPa, and a designed annual transmission capacity of 100 × 10^4^ tons. The pipeline is laid underground, and the terrain along the line is mainly desert and Gobi, with a flat surface. The parameters of the pipeline foundation are shown in Table 1.

### 3.2. Basic Equations

Based on the continuity equation, motion equation, and energy equation, a hydrodynamic and thermodynamic calculation model for supercritical CO_2_ pipelines is derived and established. The PR equation is combined, and thermodynamic relations are used for calculation. The basic differential equation system of gas pipe flow is shown in Formulas (1)–(3), and density, internal energy, and enthalpy can be expressed by pressure and temperature, respectively, as shown in Formulas (4)–(6). The six equations include the six unknown functions *p*, *T*, *ρ*, *w*, *u*, *h*. From the perspective of finding the general solution of differential equations, the system of equations is closed.

(1) $$A\frac{\partial \rho }{\partial t}+\frac{\partial }{\partial x}(A\rho w)=0$$

(2) $$A\frac{\partial (\rho w)}{\partial t}+A\frac{\partial p}{\partial x}+\frac{\partial (A\rho w^{2})}{\partial x}=-Ag\rho \frac{ds}{dx}-\frac{\lambda }{d}\frac{w^{2}}{2}\rho A$$

(3) $$-\frac{\partial Q}{\partial x}=A\frac{\partial }{\partial t}[\rho (u+\frac{w^{2}}{2}+gs)]+\frac{\partial }{\partial x}[A\rho w(h+\frac{w^{2}}{2}+gs)]$$

(4) ρ=ρ(p,T)

(5) u=u(p,T)

(6) h=h(p,T)

In the formulas, *p* denotes the absolute pressure of the fluid, in MPa; *T* denotes the absolute temperature of the fluid, in K; *ρ* denotes the fluid density, in kg/m^3^; *w* denotes the fluid velocity, in m/s; *A* denotes the cross-sectional area of the pipeline, in m^2^; *u* denotes the specific internal energy of the fluid per unit mass, in kJ/kg; *h* denotes the specific enthalpy of the fluid per unit mass, in kJ/kg; *x* denotes the distance from the starting point of the pipe section, in m; *t* denotes the time describing the flow process, in s; s denotes the elevation at each cross-section of the pipe section, in m; *g* denotes the gravitational acceleration, in m/s^2^; *λ* denotes the hydraulic friction coefficient of the pipe section; and *Q* denotes the heat dissipation rate from the fluid inside the pipe to the surrounding environment over the [0, *x*] pipe section, in kJ/s.

### 3.3. Model Building

**Figure 1:**
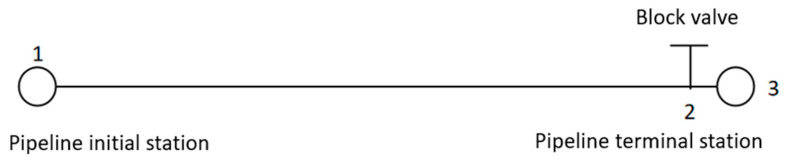
Schematic diagram of the pipeline model.

**Figure 2:**
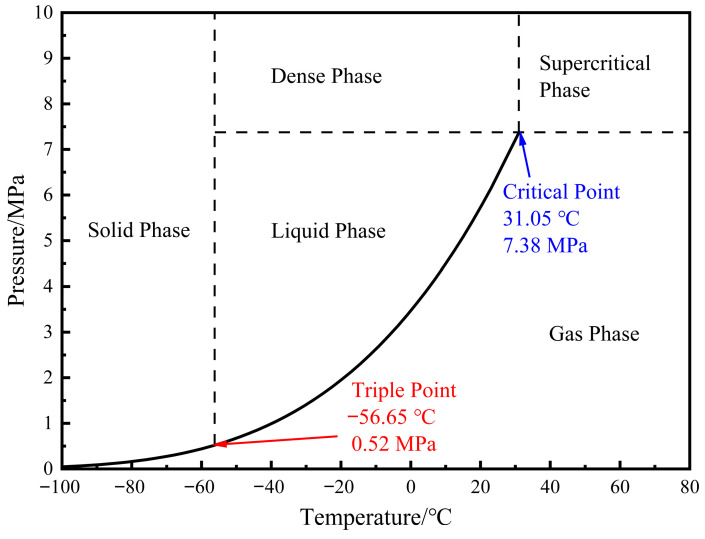
CO_2_ phase diagram.

The model is simplified to consist of an initial station (the starting point), a terminal station (the ending point), a pipeline, and a block valve located at the terminal station, as shown in Figure 1. Node 1 is the pipeline initial station, Node 2 is the block valve, and Node 3 is the pipeline terminal station. Within the range of pipeline transportation pressure and temperature, CO_2_ may exhibit multiple phase states, including a gas phase, liquid phase, dense phase, supercritical phase, and solid phase, as illustrated in the CO_2_ phase diagram (Figure 2). The critical temperature of CO_2_ is 31.05 °C, and the critical pressure is 7.38 MPa. The actual pipeline restart condition to be simulated is described as follows: At a certain moment during pipeline shutdown, a gas–liquid two-phase state has appeared in part of the fluid inside the pipeline. At this time, the block valve at the terminal station is closed, and the restart operation is performed by injecting liquid-phase CO_2_ with a certain mass flow rate at the initial station of the pipeline. With the injection of the new fluid, the mass and density of CO_2_ inside the pipeline continuously increase, accompanied by changes in pressure, temperature, and phase state at various points along the line. The pressurization stage of the pipeline ends when the pressure at the initial station reaches 12 MPa. Subsequently, supercritical CO_2_ is injected at the initial station, and the block valve at the terminal station is opened simultaneously. The newly injected supercritical CO_2_ displaces the fluid inside the pipeline out of the system, thereby completing the pipeline restart operation.

For the transient restart process of supercritical CO_2_ pipelines, the specific variation laws of the flow state (i.e., the evolutionary correlation characteristics of physical parameters such as temperature, pressure, density, liquid holdup, and phase state inside the pipeline with time and space) are not only governed by the continuity equation, motion equation, energy equation, state equation, internal energy equation, and enthalpy equation but also constrained by the boundary conditions and initial conditions of the pipeline system. Therefore, before conducting the transient restart simulation, it is necessary to determine the boundary conditions and initial conditions of the transient process. For the restart process, the initial conditions are defined as the state of the pipeline system at the onset of the investigated time period, whereas the boundary conditions are specified as the flow state at the system boundaries throughout the transient process. Based on the established model, the relevant physical property parameters of the supercritical CO_2_ pipeline under operating conditions are obtained through one-dimensional steady-state hydrodynamic and thermodynamic calculations, which serve as the initial conditions for the transient restart model.

The pipeline initial station adopts the injected CO_2_ temperature and mass flow rate as boundary conditions. Corresponding temperatures and mass flow rates are set separately before and after the pressure at the pipeline initial station reaches 12 MPa, with specific parameter settings as shown in Equations (7) and (8). The temperature range is 275.15–323.15 K (2–50 °C), and the mass flow rate range is 10–34.7 kg/s (30 × 10^4^–100 × 10^4^ tons/year).

(7) $$\left\{\begin{array}{l} T_{\mathrm{in}}=275.15\;K,\;t\leq t_{0}\\ T_{\mathrm{in}}=323.15\;K,\;t>t_{0} \end{array}\right.$$

(8) $$\left\{\begin{array}{l} m_{\mathrm{in}}=10\;\mathrm{kg}/s,\;t\leq t_{0}\\ m_{\mathrm{in}}=34.7\;\mathrm{kg}/s,\;t>t_{0} \end{array}\right.$$

In the equations, *T*_in_ denotes the temperature at the pipeline initial station, in K; *m*_in_ denotes the mass flow rate at the pipeline initial station, in kg/s; *t* denotes the time elapsed since the start of commissioning, in h; and *t*_0_ denotes the moment when the pressure at the initial station reaches 12 MPa (i.e., the moment when pipeline commissioning is completed and the block valve at the terminal station is to be opened for normal transportation), in h.

Before the pressure at the pipeline initial station reaches 12 MPa, the block valve at the pipeline terminal station remains closed, with the boundary condition of controlling the mass flow rate at the terminal station to zero. After the pressure at the pipeline initial station reaches 12 MPa, the pipeline terminal station adopts the boundary condition of maintaining a constant terminal pressure. Considering a pressure drop of 1 MPa along the pipeline, the terminal pressure is controlled at 11 MPa, with specific parameter settings as shown in Equation (9).

(9) $$\left\{\begin{array}{l} m_{\mathrm{out}}=0\;\mathrm{kg}/s,\;t\leq t_{0}\\ P_{\mathrm{out}}=11\;\mathrm{MPa},\;t>t_{0} \end{array}\right.$$

In the equation, *m*_out_ denotes the mass flow rate at the pipeline terminal station, in kg/s, and *P*_in_ denotes the pressure at the pipeline terminal station, in Pa.

### 3.4. Model Solving

**Figure 3:**
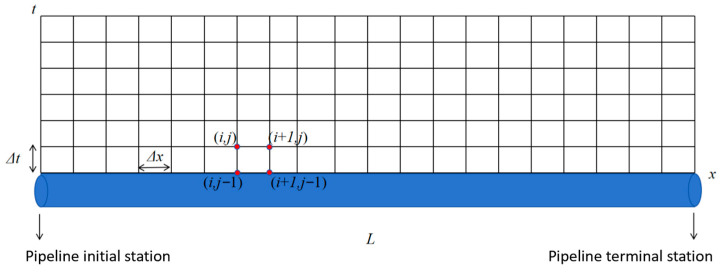
Model solving the grid diagram.

An *x*-*t* grid diagram was constructed, as shown in Figure 3, where the horizontal direction is the *x*-axis (pipeline mileage axis), the vertical direction is the *t*-axis (time axis), *L* is the total length of the pipeline, the pipeline is uniformly divided into n sections, *t*_0_ is the duration of the restart transient process, Δ*x* is the length of the small sections of the pipeline generated after the pipeline is cut, and Δ*t* is the time difference.

Before *t*_0_, the end valve is closed. The starting point, the valve, and the pipeline between them form a system in which the number of unknown parameters is 6n. And each computing grid can be composed of three difference equations, from which 3n difference equations can be obtained. There are 3 (n − 1) initial conditions at the intersection nodes of pipe sections. Adding the temperature at the starting point, the mass flow boundary condition, and the mass flow boundary condition of the valve at the end point, there are a total of 3n boundary conditions. At this point, the number of equations is (3n + 3n), and the number of unknown variables is 6n. The number of equations is equal to the number of unknown variables, and this difference equation system is solvable.

After time *t*_0_, the end valve opens, and the starting point, the end point, and the pipeline between them form a system, in which the number of unknown parameters is 6n. And each computing grid can be composed of three difference equations, from which 3n difference equations can be obtained. There are 3 (n − 1) initial conditions at the intersection nodes of the pipe sections. Adding the temperature and mass flow boundary conditions at the starting point and the pressure boundary conditions at the ending point, there are a total of 3n boundary conditions. At this point, the number of equations is (3n + 3n), and the number of unknown variables is 6n. The number of equations is equal to the number of unknown variables, and this difference equation system is solvable.

It can be seen from this that, for the two stages of the pipeline restart transient process, (1) the terminal valve remains closed, and the liquid phase CO_2_ is injected at the initial station, and (2) the terminal valve remains open, and supercritical phase CO_2_ is injected at the initial station. The difference equations are all solvable. Finally, the difference equation system is solved by using the Newton–Rapshon algorithm and the method of temporal recursion.

### 3.5. Model Validation

Based on the engineering case parameters in Section 3.1, a pipeline shutdown and restart model was established using MATLAB (v.R2021b) programming, and the calculation results were compared with those from the commercial software OLGA (v.2020.1.0). Under the pipeline operating condition with a designed throughput of 34.7 kg/s (100 × 10^4^ tons/year), the outlet pressure and temperature at the initial station are 12 MPa and 50 °C, respectively, while the inlet pressure and temperature at the terminal station are 11 MPa and 33 °C, respectively. After the pipeline is shut down for 5.20 h, the pressure and temperature along the pipeline both decrease. Taking the outlet parameters of the initial station as an example, the pressure drops to 6.8 MPa, the temperature drops to 27.6 °C, and the liquid holdup of the CO_2_ fluid decreases to 0.89, indicating that the CO_2_ has transformed from the supercritical phase to the gas–liquid two-phase state. It should be noted that liquid holdup refers to the ratio of the mass of liquid-phase (or dense-phase, or supercritical-phase) CO_2_ at a certain cross-section in the gas–liquid two-phase flow inside the pipeline to the total mass of the pipeline at that cross-section. It is a core parameter characterizing the vaporization degree of CO_2_ in the pipeline, with a variation range of 0–1. If the liquid holdup is less than one, it indicates that part of the CO_2_ in the pipeline has transformed into the gas phase, and the smaller the value, the higher the vaporization degree.

The pipeline operating condition after 5.20 h of shutdown was taken as the initial condition for pipeline restart. The restart process simulation was carried out by controlling the mass flow rate of the newly injected liquid-phase CO_2_ at the initial station to 10 kg/s and the temperature to 2 °C. It should be noted that the set value of the liquid-phase CO_2_ mass flow rate here is an assumed value, and the specific value will be discussed in Section 6.2. On the premise of not affecting the simulation accuracy, to accelerate the simulation speed, the length segment size was set to 500 m and the time step to 60 s during the simulation.

**Figure 4:**
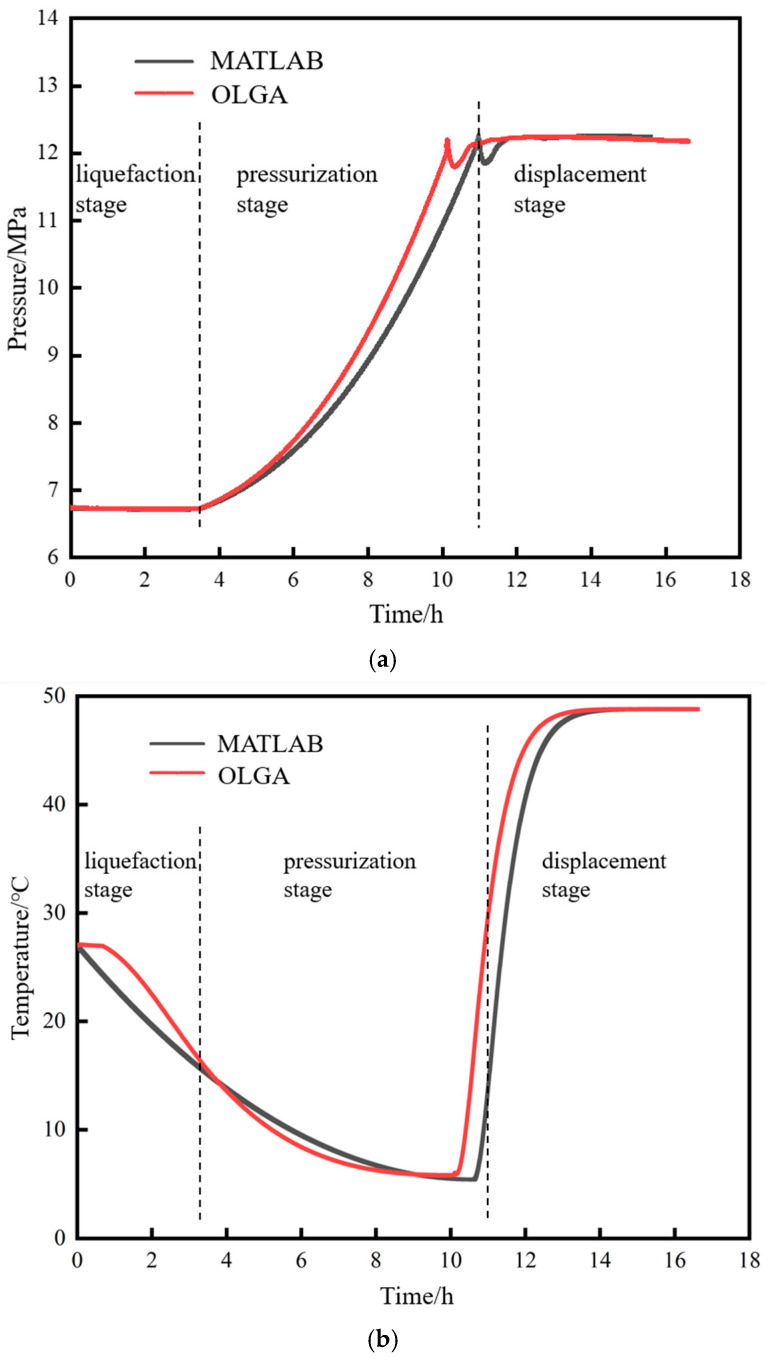

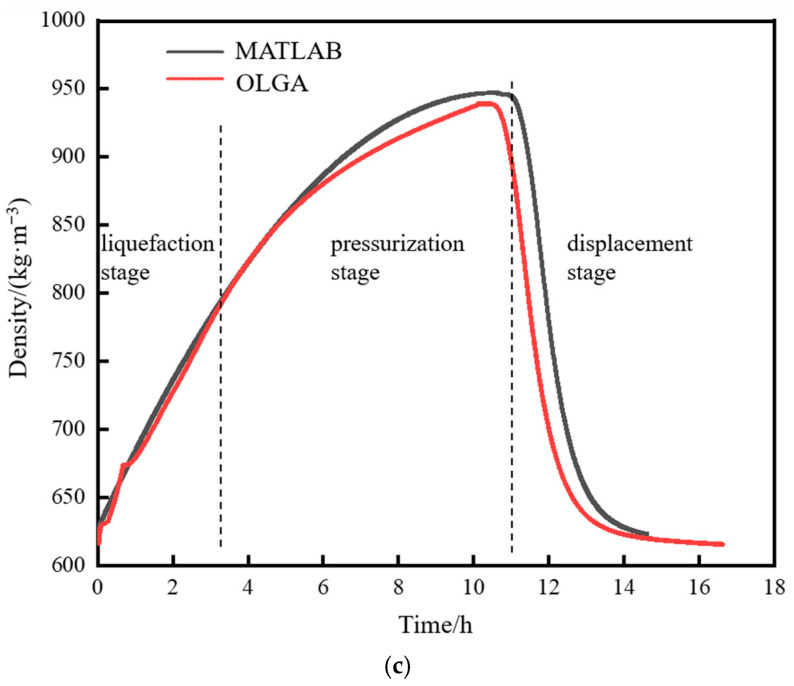
Comparison of the results of OLGA and MATLAB programming during the restart process. (**a**) Pressure. (**b**) Temperature. (**c**) Density.

The comparison results of the calculated pressure, temperature, and density at the pipeline starting point during the restart process are shown in Figure 4. The variation trends of each parameter calculated by both methods are consistent. As can be seen from Figure 4a, the restart process can be divided into three stages: a liquefaction stage, a pressurization stage, and a displacement stage. In the 3 h after liquid-phase CO_2_ is injected from the initial station, the starting point pressure remains unchanged at around 6.8 MPa, which is attributed to the CO_2_ being in a gas–liquid two-phase state throughout this period. It is not until the 3.85th hour that all gaseous CO_2_ at the starting point is transformed into the liquid phase, after which the pressure begins to rise rapidly. When the pressure rises to 12 MPa, 50.0 °C supercritical CO_2_ is injected. Due to the significant difference between the density of the newly injected 50.0 °C supercritical CO_2_ (584 kg/m^3^) and the density of the liquid-phase CO_2_ inside the pipeline (950 kg/m^3^), a sudden pressure rise followed by a sudden drop is observed in both the MATLAB and OLGA calculation results.

As can be seen from Figure 4b, since the temperature of the newly injected liquid-phase CO_2_ (2 °C) is lower than the temperature of the gas–liquid two-phase flow at the starting point (27 °C), the temperature of the fluid at the starting point continuously decreases. It is not until the injection of high-temperature supercritical CO_2_ that the fluid temperature begins to rise continuously, eventually returning to 50 °C, which is the temperature before the pipeline shutdown. As can be seen from Figure 4c, due to the phase transition of the fluid at the starting point from the gas–liquid two-phase → liquid phase → dense phase → supercritical phase, the density first increases continuously. It is not until the injection of high-temperature supercritical CO_2_ that the fluid density starts to decrease.

In each stage of the restart process (liquefaction, pressurization, and displacement), the errors of pressure, temperature, and density calculated by MATLAB programming are controlled within 2%, indicating that the established restart model in this paper is feasible in terms of accuracy.

## 4. The Variation Law of Parameters During the Process of Pipeline Shutdown and Restart

### 4.1. Pipeline Operating Parameters Under Normal Conditions

**Figure 5:**
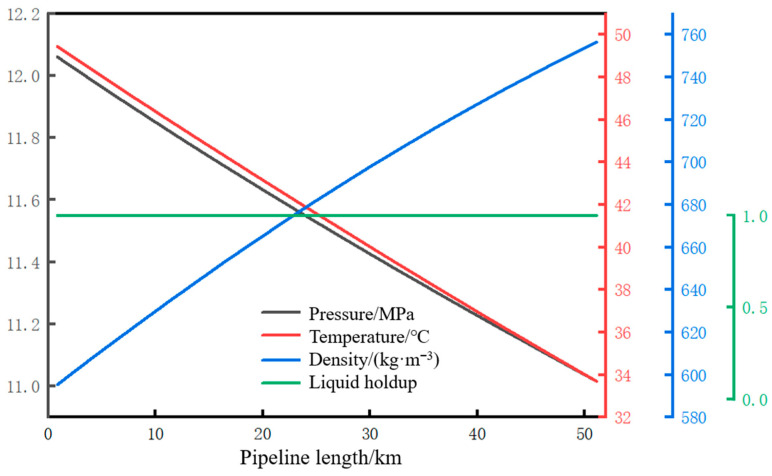
Along the path of each parameter during normal operation of the pipeline.

The distribution of pressure, temperature, liquid holdup, and density along the pipeline during normal operation is shown in Figure 5. With a pipeline throughput of 34.7 kg/s and an ambient temperature of 2 °C, the calculated pressures at the initial station and the terminal station are 12 MPa and 11 MPa, respectively. The temperatures are 50 °C and 33.7 °C, respectively, and the densities are 595 kg/m^3^ and 757 kg/m^3^, respectively. Under the current operating conditions, the fluid inside the pipeline is in a supercritical state, and the liquid holdup of the fluid at each node along the pipeline is one.

### 4.2. Analysis of Pipeline Shutdown Process

#### 4.2.1. Parameter Changes Along the Entire Pipeline During Shutdown

**Figure 6:**
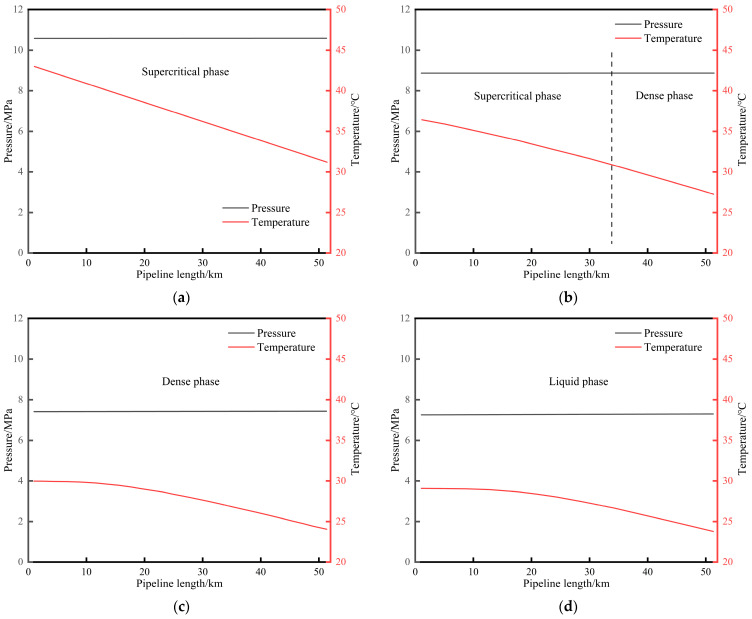

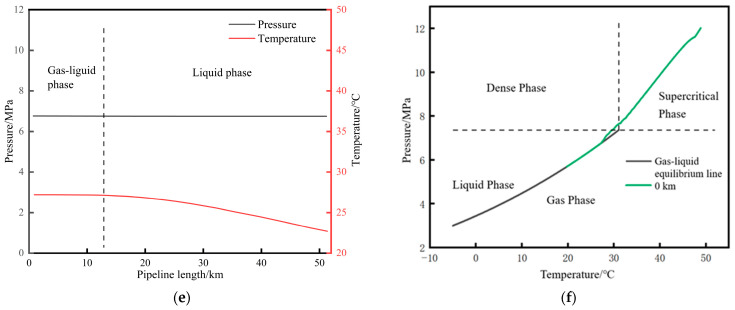
Diagram of CO_2_ temperature, pressure, and phase state changes during the pipeline shutdown process. (**a**) 1.83 h after shutdown. (**b**) 3.50 h after shutdown. (**c**) 4.91 h after shutdown. (**d**) 5.00 h after shutdown. (**e**) 5.41 h after shutdown. (**f**) Phase migration diagram of fluid at the starting point of pipeline.

After the pipeline shutdown, the pressure and temperature at various points along the pipeline decrease synchronously. The changes in temperature, pressure, and phase state of CO_2_ during the pipeline shutdown process are shown in Figure 6. At 1.83 h, the fluid along the entire line is in a supercritical state. At 3.5 h, the fluid in the downstream pipe section transforms into a dense phase. At 4.91 h and 5 h, the fluid along the entire line is in a dense phase and a liquid phase, respectively. At 5.41 h, the fluid in the upstream pipe section transforms into a gas–liquid two-phase state. Taking the fluid at the initial station outlet as an example, the phase transition path is supercritical phase ⟶ dense phase ⟶ liquid phase ⟶ gas–liquid two-phase.

#### 4.2.2. Derivation and Validation of the Liquid Holdup Calculation Formula

When fluid vaporization occurs, the pressure–temperature state point migrates along the gas–liquid equilibrium line. The key focus of the analysis is to clarify the relationship between liquid holdup and pressure–temperature during pipeline shutdown after fluid vaporization. To further reveal the variation law of liquid holdup during pipeline shutdown and achieve an accurate prediction of the pipeline shutdown state, this section presents a calculation formula for liquid holdup changes after vaporization.

After the pipeline shutdown, both the mass and volume of fluid inside the pipeline remain unchanged. Therefore, the average density of the fluid during pipeline shutdown will always be consistent with the average density before shutdown. After the fluid phase transition occurs, the pressure–temperature state point of the gas–liquid mixture moves along the gas–liquid equilibrium line, and the pressure and temperature continuously decrease. To maintain a constant average density of the gas–liquid mixture, the fluid liquid holdup will change. Thus, the liquid holdup under different saturation pressures and temperatures during the movement of the pressure–temperature state point along the gas–liquid equilibrium line can be calculated based on the density of the single-phase CO_2_ fluid before shutdown and the densities of gaseous and liquid CO_2_ under different saturation pressures and temperatures.

Let *ρ*_0_ be the density of the single-phase CO_2_ fluid before shutdown, *ρ*_g_ and *ρ*_L_ be the densities of gaseous and liquid CO_2_ under saturation pressure and temperature, respectively, and *h*_L_ be the liquid holdup under the corresponding state. Based on Equation (10), the calculation method for liquid holdup under saturation pressure and temperature is derived as shown in Equation (11).

(10) $$\rho_{0}=\rho_{L}h_{L}+\rho_{g}\left(1-h_{L}\right)$$

(11) $$h_{L}=\frac{\rho_{0}-\rho_{g}}{\rho_{L}-\rho_{g}}$$

According to Equation (11), the liquid holdup under different pressures and temperatures can be predicted. Taking the initial operating condition of 10.5 MPa and 40.2 °C as an example, the calculated results of gaseous and liquid CO_2_ densities and the predicted liquid holdup under different pressures and temperatures during shutdown are listed in Table 2.

To verify the accuracy of the proposed calculation formula for liquid holdup changes after fluid vaporization, the calculation results of the formula (calculated values) are compared with the simulation results of the OLGA software (simulated values). Table 3 presents the comparison between the calculated and simulated liquid holdup values during shutdown after fluid vaporization under different saturation pressures and temperatures. The error between the calculated and simulated liquid holdup values after fluid vaporization is within ±4%, indicating that the proposed formula has high accuracy and can accurately predict the changes in pipeline liquid holdup after vaporization occurs.

### 4.3. Pipeline Parameters Along the Line at the Initial Moment of Restart

**Figure 7:**
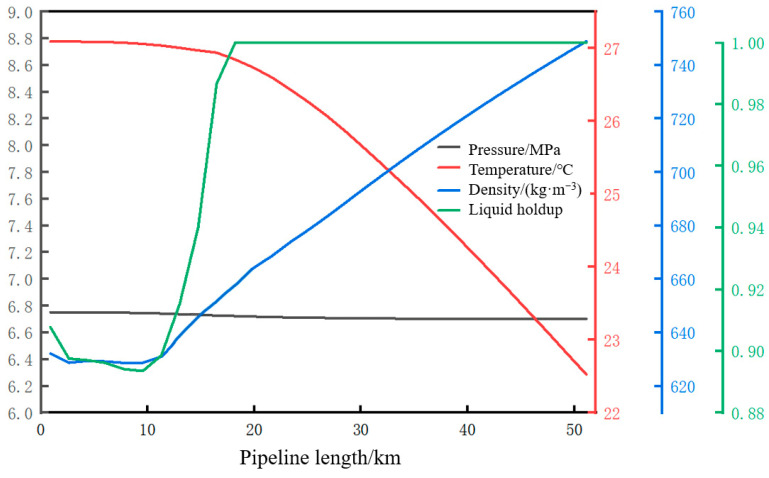
Trajectory diagram of parameters at the initial moment of pipeline restart.

The pipeline shutdown is simulated. Due to the temperature difference between the inside and outside of the pipeline and the synergistic variation relationship between CO_2_ temperature and pressure, the pressure and temperature of the CO_2_ fluid along the entire line continuously decrease. The phase transition of the fluid first occurs at the initial station and then continuously extends toward the terminal. After 5.41 h of shutdown, the distribution of pressure, temperature, liquid holdup, and density along the pipeline is shown in Figure 7, and this moment is taken as the initial moment of pipeline restart. At this point, the pressure along the entire line is stable at around 6.75 MPa; the temperatures at the initial station and terminal station are 27.1 °C and 22.5 °C, respectively; and the densities at the initial station and terminal station are 630 kg/m^3^ and 750 kg/m^3^, respectively. The 18 km upstream pipe section presents a gas–liquid two-phase state with a liquid holdup range of 0.89–1; the remaining pipe sections present a single liquid phase with a liquid holdup of 1.

### 4.4. Analysis of the Restart Process

During the pipeline restart process, the mass flow rate of liquid-phase/dense-phase CO_2_ injected at the pipeline initial station is controlled at 10 kg/s and a temperature of 2 °C, and the inlet valve at the terminal station remains closed. When the pressure at the pipeline inlet reaches 12 MPa, the inlet valve at the terminal station is opened, and the mass flow rate of supercritical CO_2_ injected at the pipeline initial station is controlled at 34.7 kg/s with a temperature of 50 °C.

The calculation results are shown in Table 4. The liquefaction and pressurization stages take 10.10 h, requiring 363.6 tons of liquid-phase and dense-phase CO_2_. The displacement stage takes 25.49 h, requiring 3184.2 tons of supercritical CO_2_. The two stages take a total of 35.59 h, with a total injection of 3547.8 tons of liquid-phase, dense-phase, and supercritical-phase CO_2_.

#### 4.4.1. Parameter Changes Along the Entire Pipeline During the Restart Process

**Figure 8:**
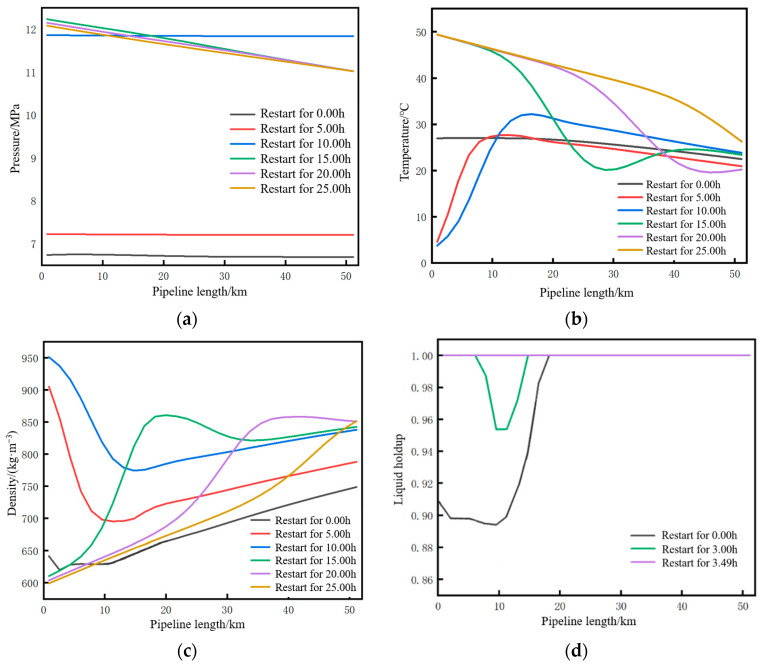
Diagram of parameters along the pipeline restart process. (**a**) Pressure. (**b**) Temperature. (**c**) Density. (**d**) Liquid holdup.

**Figure 9:**
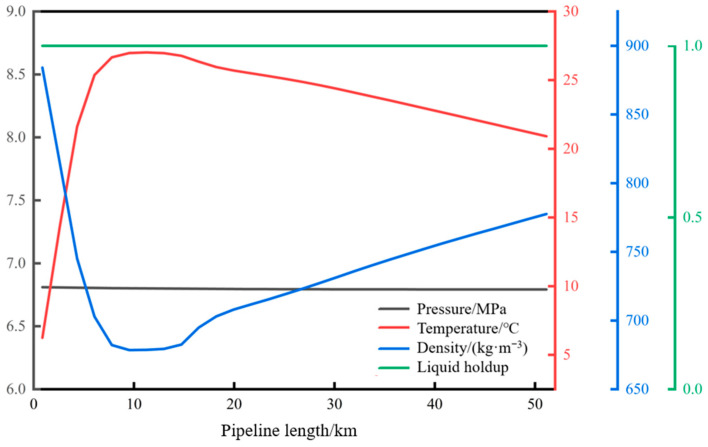
Pipeline restart 3.49 h parameter trajectory diagram.

The variation trends of pressure, temperature, density, and liquid holdup along the pipeline at different moments (0.00 h, 5.00 h, 10.00 h, 15.00 h, 20.00 h, and 25.00 h) after the pipeline restart are shown in Figure 8. As can be seen from Figure 8a, at the initial moment of restart, the fluid pressure along the entire pipeline tends to be consistent and stabilizes at 6.75 MPa. From Figure 8d, at the initial moment of restart, the 18 km upstream pipe section has undergone varying degrees of vaporization with a liquid holdup of 0.89–1. With the injection of liquid-phase CO_2_ at the initial station, liquefaction first occurs at both ends of the original gas–liquid two-phase pipe section. The length of the gas–liquid two-phase pipe section continuously decreases, and the liquid holdup continuously increases. At 3.49 h, the liquid holdup of the entire line reaches one, indicating that all gaseous CO_2_ in the 18 km upstream pipe section has completed the liquid-phase transformation. The parameters along the pipeline at 3.49 h are shown in Figure 9. The pressure along the entire line remains stable at 6.75 MPa, indicating that the pressure remains constant during the transition from the gas phase to the liquid phase.

#### 4.4.2. Parameter Changes at Characteristic Points During the Pipeline Restart Process

**Figure 10:**
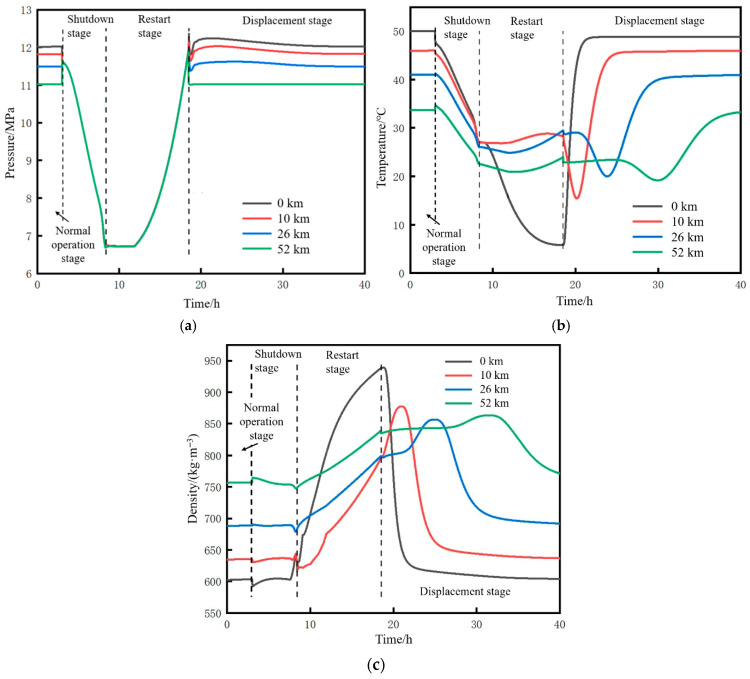
Time trend of each parameter during the pipeline restart process. (**a**) Pressure. (**b**) Temperature. (**c**) Density.

Four positions of the pipeline, namely the initial station outlet (0 km), 10 km mark, midpoint (26 km), and terminal station inlet (52 km), were selected as characteristic points to study the variation trends of temperature, pressure, and density at each point across four stages, namely normal operation, shutdown, restart, and displacement, as shown in Figure 10. The simulation results are consistent with the variation trends of the programming calculation results in Section 3.5.

According to Figure 10a, during the liquefaction stage, the pressure at each point remains constant. Once CO_2_ completes the gas–liquid transformation, the pressure rises rapidly. During the pressurization stage, the average pressure rise rate of the pipeline is 0.79 MPa/h. Ultimately, the pressure at each characteristic point returns to the pressure before shutdown. As shown in Figure 10b, during the liquefaction and pressurization stages, the temperature at the pipeline initial station continuously decreases from 27.1 °C to 5.0 °C due to the influence of newly injected low-temperature CO_2_, while the temperature at other points changes slightly within the range of 20–30 °C. From Figure 10c, the density at the initial station exhibits the largest variation range.

**Figure 11:**
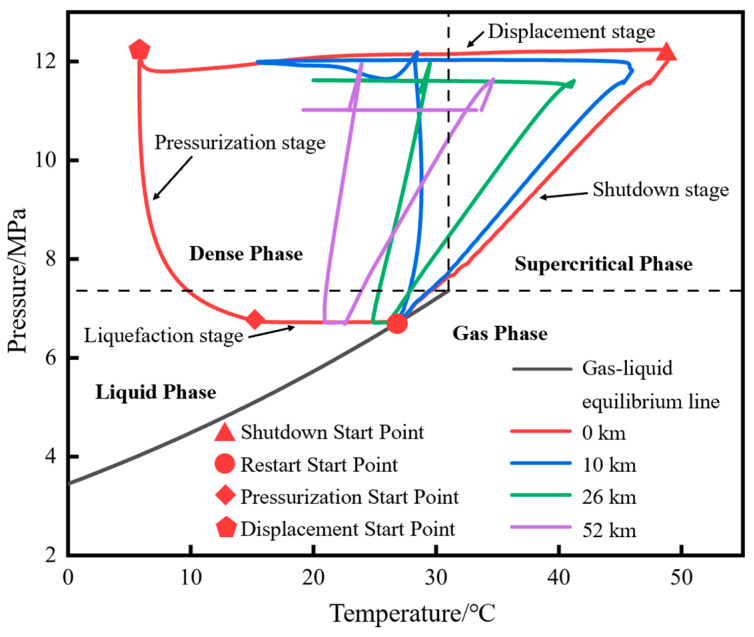
Diagram of phase point transitions of characteristic points during shutdown and restart processes.

The phase migration paths of each characteristic point during the shutdown and restart processes are marked on the phase diagram, as shown in Figure 11. The phase migration path of each characteristic point can be closed to form a closed curve, approximately trapezoidal. The closer to the initial station, the larger the envelope area of the migration path. From the initial station to the terminal station, the envelope area continuously shifts to the left. Taking the initial station as an example: before shutdown, the pressure and temperature of supercritical CO_2_ are 12 MPa and 50 °C. During the shutdown stage, due to the simultaneous decrease in temperature and pressure of the fluid inside the pipeline, its phase migration path moves from the initial point to the lower left in the supercritical phase region. After entering the liquid phase region, it further reaches the gas–liquid equilibrium line and continues to move toward low temperature and low pressure along the line. When the fluid pressure and temperature are 6.75 MPa and 27.1 °C, respectively, the restart operation is performed. Affected by the temperature of the newly injected low-temperature fluid, and in the early stage of restart when the fluid at the initial station is transforming from a gas–liquid two-phase to a liquid phase, the fluid temperature continuously decreases, but the pressure remains nearly stable. This is reflected in the phase diagram as a leftward horizontal movement trend of the phase migration path. When liquefaction is completed, the fluid pressure and temperature are 6.75 MPa and 14.8 °C, respectively. The fluid pressure inside the pipeline gradually rises, and the phase migration path enters the dense-phase region. Before entering the supercritical phase, there are significant differences in the duration of each phase state: the gas–liquid two-phase lasts for 0.67 h, the liquid phase for 4.68 h, and the dense phase for 5.69 h. When the pressure rises to 12 MPa, the displacement operation is carried out. Due to the opening of the terminal station valve, the pressure at the initial station no longer continues to rise, while the temperature keeps increasing. At this point, the phase migration path enters the supercritical phase region and eventually returns to the operating point before shutdown. The entire shutdown and restart process can actually be further subdivided into four stages: a shutdown stage, a liquefaction stage, a pressurization stage, and a displacement stage. The four sides of the trapezoid can be defined as the shutdown line, liquefaction line, pressurization line, and displacement line, respectively. The four vertices can be defined as the shutdown initial point, shutdown completion point (restart initial point), liquefaction completion point (pressurization start point), pressurization completion point (displacement start point), and displacement completion point (normal operation point).

## 5. Shutdown and Restart Experiment

### 5.1. Experimental Facility

**Figure 12:**
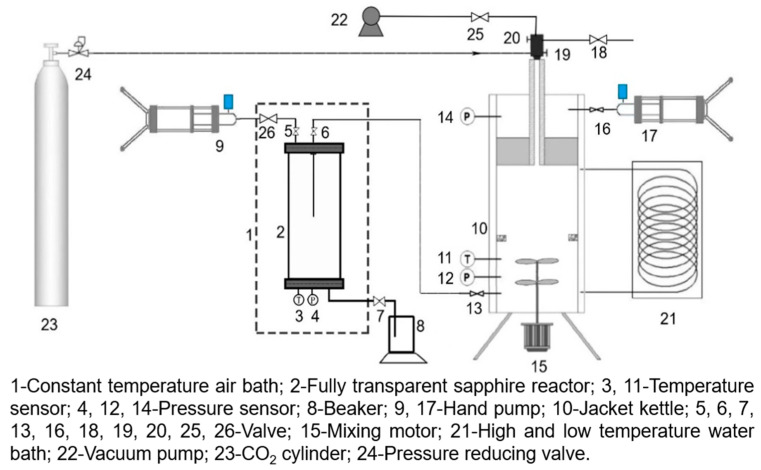
Schematic diagram of the high-pressure visual CO_2_ phase analysis system.

**Figure 13:**
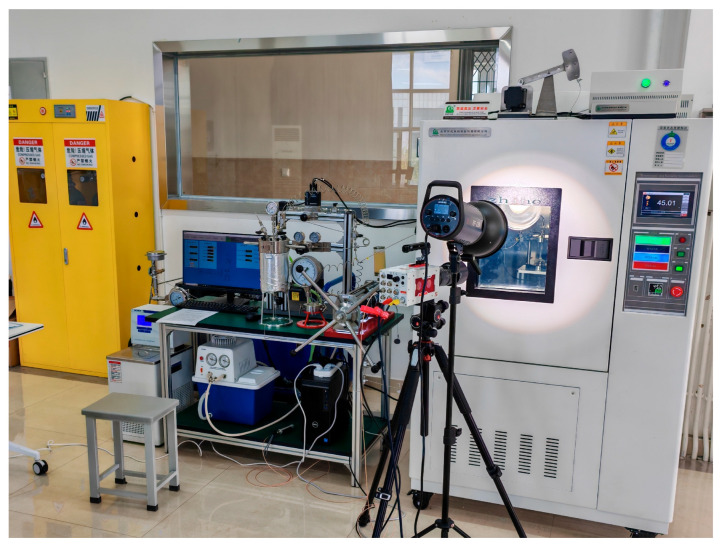
Physical diagram of the high-pressure visual CO_2_ phase analysis system.

A self-built high-pressure visual CO_2_ phase analysis system was adopted to experimentally simulate changes in parameters such as pressure, temperature, liquid holdup, and phase state during the pipeline restart process. The experimental system includes a fully transparent sapphire reactor, jacketed reactor, constant temperature air bath, high- and low-temperature water bath, high-speed camera, temperature sensor, pressure sensor, data acquisition system, etc., as shown in Figure 12 and Figure 13. The initial state of CO_2_ in the pipeline, meeting the requirements of test pressure, temperature, phase state, and liquid holdup, was prepared in the sapphire reactor using CO_2_ gas cylinders, compressors, fully transparent sapphire reactors, constant temperature air baths, and other equipment. The pipeline shutdown process was simulated by controlling the temperature of the constant temperature air bath. Through experimental devices such as CO_2_ gas cylinders, compressors, jacketed reactors, and high- and low-temperature water baths, CO_2_ of different phases meeting the test pressure and temperature requirements was prepared in the jacketed reactor as the newly injected medium for the restart process. The jacketed reactor was connected to the sapphire reactor flow to realize the extrusion of the shutdown medium by the newly injected medium in the sapphire reactor, thereby simulating the real pipeline restart process. The pressure, temperature, liquid holdup, and phase state changes of the medium in the sapphire reactor during shutdown and restart were observed and recorded, and the moment of phase state change was captured using a high-speed camera.

### 5.2. Experimental Conditions

The specific experimental conditions are as follows:

(1) Shutdown stage: CO_2_ fluid with a temperature of 40.2 °C and a pressure of 10.41 MPa was prepared in the sapphire reactor, and the temperature of the constant temperature box was rapidly reduced to 2 °C to simulate the pipeline shutdown process in winter. The changes in pressure, temperature, and phase state over time were recorded as the liquid holdup of CO_2_ in the reactor decreased from 1.0 to 0.9, 0.8, and 0.7;

(2) Restart liquefaction stage: The operating condition with a fluid liquid holdup of 0.7 in the pipeline was taken as the initial condition for pipeline restart. Liquid-phase CO_2_ with a pressure of 6.0 MPa and a temperature of 2 °C was prepared in the jacketed reactor. The jacketed reactor was connected to the sapphire reactor flow, and the high-pressure liquid-phase CO_2_ in the jacketed reactor was injected into the sapphire reactor to simulate the pipeline restart process. The changes in pressure, temperature, and phase state over time were recorded as the liquid holdup of CO_2_ in the reactor increased from 0.7 to 0.8, 0.9, and 1.0.

### 5.3. Experimental Results

#### 5.3.1. Shutdown Process

**Figure 14:**
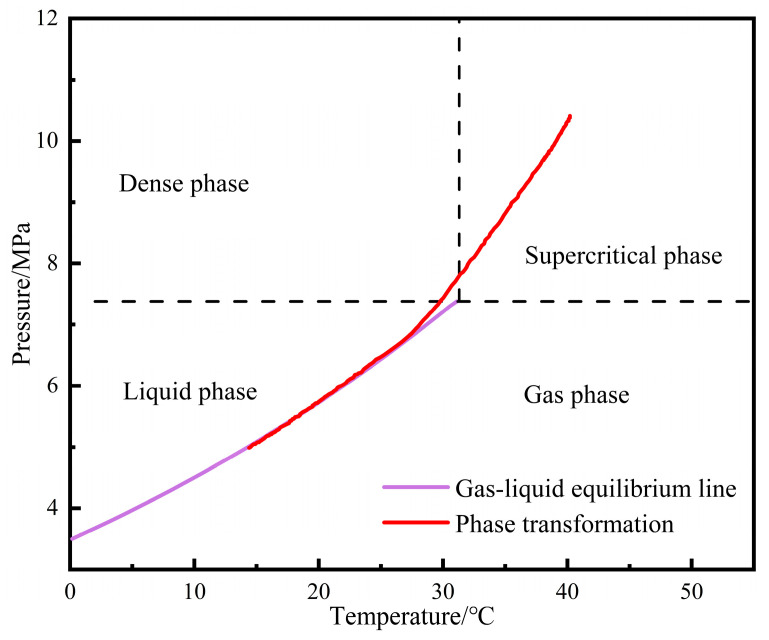
Phase change diagram of fluid during the shutdown process.

The phase migration path during the entire shutdown process is shown in Figure 14. The phase migration path was supercritical phase → dense phase → liquid phase → gas–liquid two-phase, which is consistent with the simulation results presented in Section 4.2.1 above. The parameter changes at different shutdown times are shown in Table 5. At 588 s, the pressure of the CO_2_ fluid dropped to 6.66 MPa, the temperature dropped to 26.30 °C, and the liquid holdup was 0.9.

**Figure 15:**
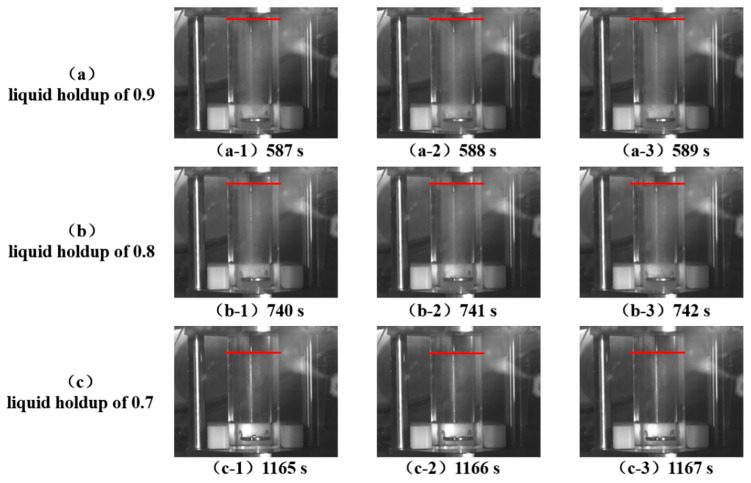
Gas–liquid stratification in the reactor when the shutdown liquid holdup reaches 0.9, 0.8, and 0.7. The red line indicates the liquid level.

The gas–liquid stratification in the reactor at different liquid holdups (0.9, 0.8, and 0.7) during CO_2_ fluid shutdown was recorded, as shown in Figure 15. The upper part of the reactor was gas, and the lower part was liquid. With the extension of shutdown time, the liquid holdup decreased, and the gas–liquid interface moved downward. When the CO_2_ fluid was shut down to a pressure of 5 MPa and a temperature of 14.4 °C, the liquid holdup was 0.7.

#### 5.3.2. Restart Process

**Figure 16:**
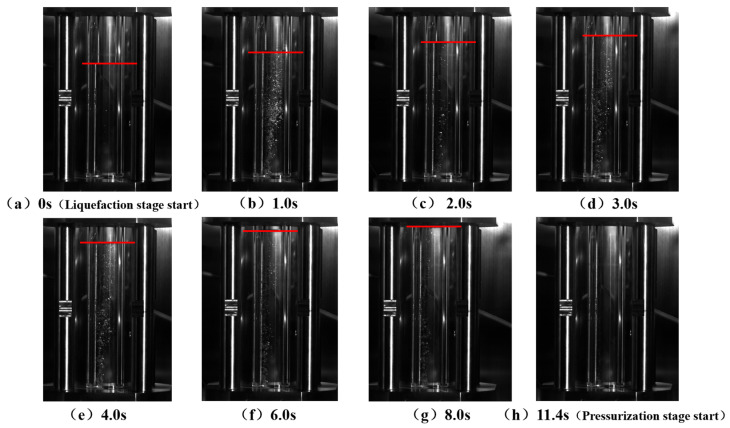
Experimental CO_2_ liquid holdup change diagram during the restart process. The red line indicates the liquid level.

The experimental results of the restart stage are shown in Figure 16. At time 0 of restart, the liquid holdup of the CO_2_ fluid in the reactor was 0.7, with liquid at the bottom and gas at the top. After new liquid was injected from the bottom of the reactor, the existing fluid was squeezed, the pressure in the reactor increased, part of the gaseous CO_2_ fluid was liquefied, and the fluid liquid holdup continuously increased. At 8 s, the liquid holdup reached one, and all gaseous CO_2_ was completely liquefied. This experiment verified that the gas–liquid two-phase CO_2_ in the pipeline can be transformed into the liquid phase through pressurization during the pipeline restart process.

**Figure 17:**
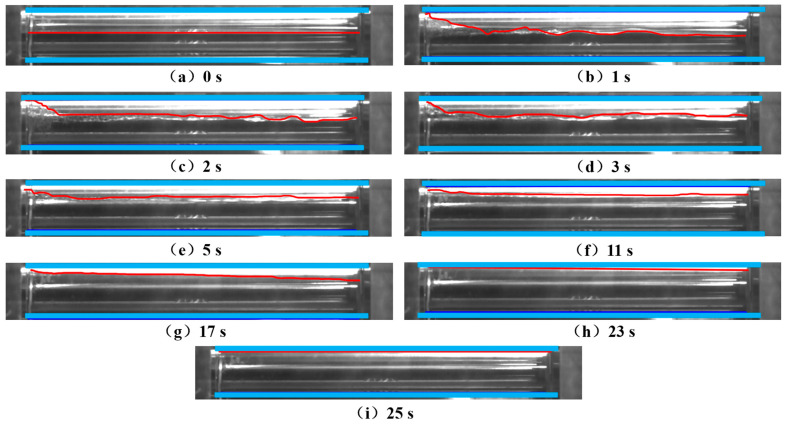
Change diagram of the axial CO_2_ gas–liquid interface in the sapphire reactor during the restart process. The red line indicates the liquid level, and the blue line denotes the boundary of sapphire reactor.

To make the experiment more consistent with the actual pipeline operating conditions, the sapphire reactor was placed horizontally. The leftmost side of the sapphire reactor was used as the pipeline initial station, the newly injected fluid from the jacket kettle flowed into the sapphire reactor from the left side, and the right side of the sapphire reactor was used as the pipeline terminal station (closed end). The restart process of the pipeline shut down to the same operating condition as above (pressure 5 MPa, temperature 14.4 °C, liquid holdup 0.7) was simulated, and the changes in the gas–liquid interface at different axial positions of the sapphire reactor at different times were recorded, as shown in Figure 17.

The upper blue line and lower blue line represent the top and bottom of the reactor, respectively, and the middle red line represents the gas–liquid interface. It can be seen that, at the initial moment, the gas–liquid interface was a horizontal line, indicating that the liquid holdup was consistent at different axial positions of the reactor. After the new fluid was injected from the left side of the reactor, the gaseous CO_2_ at different axial positions of the reactor began to liquefy at different times. The closer to the new fluid injection side, the earlier the liquefaction occurred. For example, at 17 s, the gas–liquid interface became an inclined line, the CO_2_ liquefaction degree in the left region was higher, and the liquefaction degree in the rear region was relatively lower. At 25 s, the gaseous CO_2_ in the reactor was completely transformed into the liquid phase. At this time, the fluid pressure was 5.2 MPa, and the temperature was 2.7 °C. The experimental results are consistent with the simulation results presented in Section 4.4.1 above.

## 6. Analysis of Influencing Factors in the Restart Process

When the pipeline inevitably exceeds its safe shutdown time, the fluid inside the pipe will undergo a phase transition from the supercritical phase to the gas–liquid two-phase state, necessitating the formulation of safety control measures for the restart process. Through simulations, it was found that the key influencing factors affecting the restart process, such as liquefaction time, pressure rise time, pressure rise rate, and CO_2_ consumption, may include the liquid holdup of the fluid in the pipe at the initial moment of restart, the mass flow rate of CO_2_ injected at the initial station during the restart process, and the soil temperature around the pipeline. To further analyze the degree of influence of these key factors and provide reference control indicators for the development of restart measure plans, an analysis of the influencing factors in the restart process was conducted.

The values of different influencing factors are shown in Table 6. The liquid holdup was set to 0.5, 0.6, 0.7, 0.8, and 0.9, respectively. The medium injection flow rates at the initial station were set to 4.0 kg/s, 10.0 kg/s, 14.0 kg/s, 26.0 kg/s, 30.0 kg/s, and 34.7 kg/s, respectively. The ambient temperatures were set to 2 °C, 12 °C, and 22 °C, corresponding to the ground temperature conditions in winter, average year, and summer, respectively.

### 6.1. Liquid Holdup

**Figure 18:**
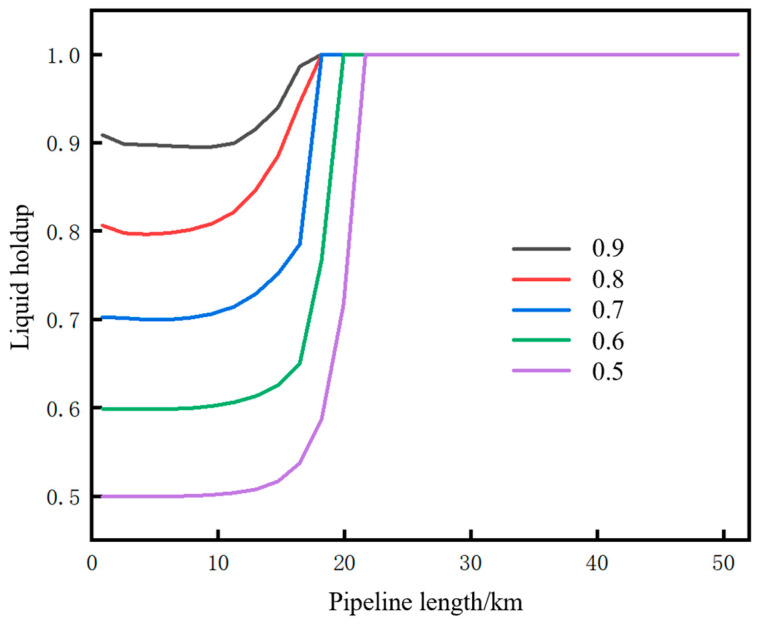
Distribution of liquid holdup along the pipeline after different shutdown durations.

When the pipeline operates under different shutdown time conditions, the liquid holdup of the fluid inside the pipe varies, and the calculation results are shown in Table 7 and Figure 18. Vaporization first occurs at the pipeline initial station. With the extension of shutdown time, the length of the pipe section with gas–liquid two-phase flow in the upstream of the pipeline continuously increases. Taking the fluid at the initial station outlet as an example, the liquid holdup is 0.9, 0.8, 0.7, 0.6, and 0.5 after shutdown for 5.41 h, 6.37 h, 8.88 h, 13.75 h, and 20.9 h, respectively. Despite the significant differences in shutdown time, the pressure and temperature inside the pipe at the end of shutdown do not vary much—the pressure ranges from 6.36 MPa to 6.75 MPa, and the temperature ranges from 24.5 °C to 27.1 °C—with the phase points moving along the gas–liquid equilibrium line.

**Figure 19:**
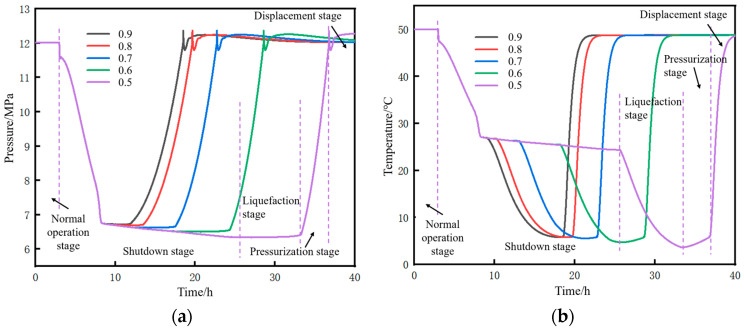
Variation diagram of parameters at the initial station during pipeline shutdown and restart under different liquid holdup conditions. (**a**) Pressure at the initial station. (**b**) Temperature at the initial station.

Based on an injection mass flow rate of 10 kg/s at the initial station and a soil temperature of 2 °C, the changes in pressure and temperature parameters at the initial station during the pipeline restart process after shutdown to different liquid holdup conditions were calculated, as shown in Figure 19. The stages displayed in the figure include operation, shutdown, liquefaction, pressurization, and displacement. For both pressure and temperature, the lower the liquid holdup, the longer the time required to return to the state before shutdown. After the completion of the pressurization stage, at the initial moment of supercritical CO_2_ displacing dense-phase CO_2_, due to the density of the newly injected supercritical CO_2_ being lower than that of the existing dense-phase CO_2_ inside the pipe (with a density difference of approximately 366 kg/m^3^), according to the principle of momentum conservation, the high-density dense-phase CO_2_ exerts a reverse resistance on the low-density supercritical CO_2_. This situation forms an instantaneous impact at the contact interface of the two fluids. The kinetic energy cannot be dissipated quickly, leading to local pressure accumulation at the contact interface, which ultimately manifests as a sudden pressure surge, as shown in Figure 19a.

**Figure 20:**
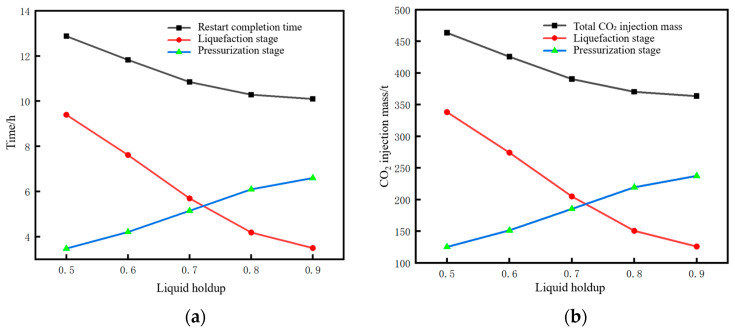
Trend diagram of time parameters and corresponding CO_2_ injection mass during pipeline restart under different liquid holdups. (**a**) Time parameters of the restart process. (**b**) CO_2_ injection mass.

The time parameters of the pipeline restart process and the corresponding changes in CO_2_ injection mass under different liquid holdups are shown in Figure 20. The restart completion time is defined as the time when the pressure at the pipeline initial station recovers to 12 MPa (the value before shutdown). The lower the liquid holdup, the longer the required restart completion time. This time consists of liquefaction time and pressurization time. As the liquid holdup decreases, the liquefaction time increases while the pressurization time decreases. However, the reduction in pressurization time is insufficient to offset the increase in liquefaction time, resulting in an overall longer duration. The reason is that, for low liquid holdup conditions, the injection time and mass of CO_2_ at the initial station during the liquefaction stage are larger. When liquefaction is completed and pressurization begins, the mass and density of CO_2_ inside the pipe are relatively high, requiring less time to pressurize to 12 MPa. For example, when the liquid holdup is 0.9, the liquefaction time is 2.1 h, the mass of CO_2_ injected during the liquefaction stage is 125 t, and the pressurization time is 6.4 h. However, when the liquid holdup is 0.5, the liquefaction time is 9.8 h, the mass of CO_2_ injected during the liquefaction stage is 342 t, and the pressurization time is 1.9 h. Both the total restart time and the total CO_2_ injection mass increase with the decrease in liquid holdup. This indicates that, if the pipeline is shut down for an excessively long time, the cost of restarting the pipeline will be greater when the liquid holdup has dropped to a relatively low level. In terms of time, a liquid holdup of 0.5 takes 2.5 h longer than that of 0.9. In terms of the CO_2_ injection mass, a liquid holdup of 0.5 requires 90 t more injection than that of 0.9.

**Figure 21:**
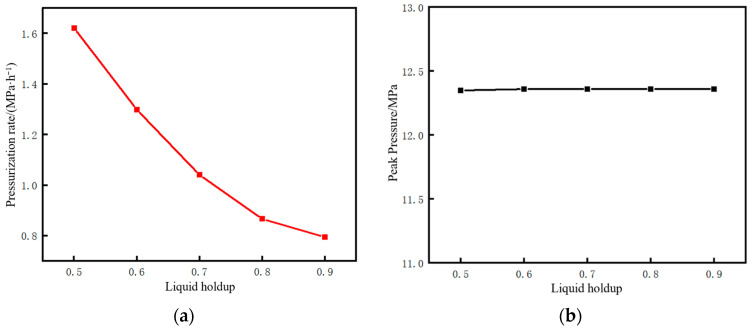
Diagram of pressure rise rate and restart pressure peak at the initial station during pipeline restart under different liquid holdups. (**a**) Pressure rise rate. (**b**) Peak pressure.

The pressure rise rate and pressure peak at the initial station during the pipeline restart process under different liquid holdups are shown in Figure 21. The correlation mechanism between the pressure rise rate and liquid holdup lies in the different cumulative masses of CO_2_ injected during the liquefaction stage. When the liquid holdup is 0.5, the total mass of injected CO_2_ is 342 t, which is 2.74 times that of 125 t when the liquid holdup is 0.9. Consequently, the pressure rise rate at a liquid holdup of 0.5 is 1.61 MPa/h, which is 2.04 times that of 0.79 MPa/h at a liquid holdup of 0.9. Under different liquid holdup conditions, the pressure peak stabilizes at 12.36 MPa, indicating that the liquid holdup has no correlation with whether overpressure occurs during pipeline restart. Zhu et al. [28] studied the variation law of process parameters during the commissioning of a supercritical CO_2_ pipeline and suggested, from the perspective of pipeline safety, that the pipeline pressure rise rate should not exceed 1 MPa/h. Based on this criterion, the liquid holdup of the pipeline in this case needs to be controlled above 0.7.

**Figure 22:**
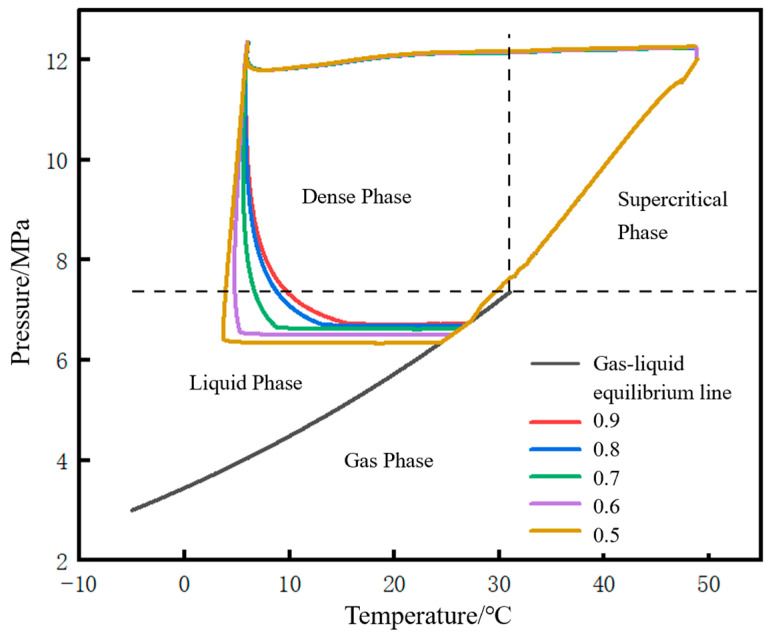
Phase point migration diagram of fluid at pipeline starting point under different liquid holdups.

The migration paths of the fluid at the initial station outlet on the CO_2_ phase diagram under different liquid holdups are shown in Figure 22. The lower the liquid holdup, the larger the envelope area of the closed curve formed by the phase migration path. The differences among different liquid holdups are mainly reflected in the liquid phase region and dense phase region. The angle between the liquefaction line and the pressurization line (defined earlier) gradually decreases as the liquid holdup decreases, and when the liquid holdup is 0.5, the angle between the two lines is nearly 90°. The reason is that the lower the liquid holdup, the faster the pressure rise rate.

### 6.2. Injection CO_2_ Mass Flow Rate

**Figure 23:**
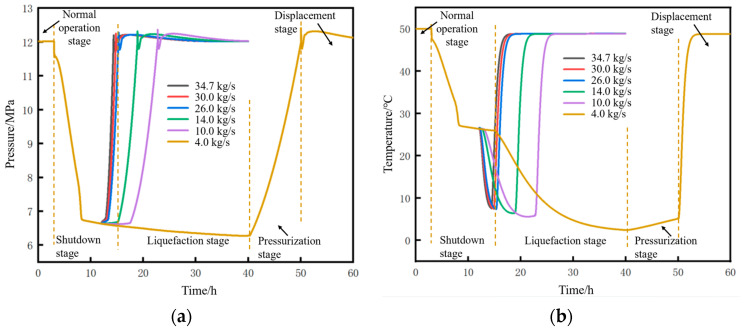
Variation diagram of parameters at the pipeline initial station outlet during pipeline shutdown and restart under different injection mass flow rates. (**a**) Pressure. (**b**) Temperature.

Based on a liquid holdup of 0.89–1 in the 18 km upstream pipe section and a soil temperature of 2 °C, the changes in parameters at the pipeline initial station during the pipeline shutdown and restart processes under different injection mass flow rates were calculated, as shown in Figure 23. For both pressure and temperature, the lower the mass flow rate, the longer the time required to return to the state before shutdown.

**Figure 24:**
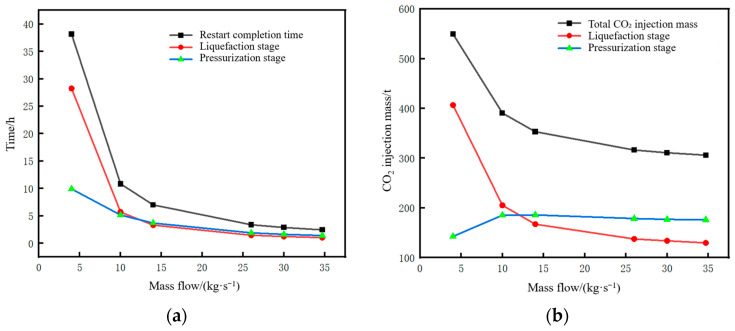
Variation diagram of time parameters and corresponding CO_2_ injection mass during pipeline restart under different injection mass flow rates. (**a**) Time parameters of the restart process. (**b**) CO_2_ injection mass.

The time parameters of the pipeline restart process and the corresponding changes in CO_2_ injection mass under different mass flow rates are shown in Figure 24. When the injected CO_2_ mass flow rate decreases below 10 kg/s, a distinct inflection point can be observed in the liquefaction time and restart completion time curves in Figure 24a. A low mass flow rate will lead to an excessively long liquefaction time, thereby increasing the restart completion time. As can be seen from Figure 24b, when the injected mass flow rate is low, the long injection time during the liquefaction process results in a very large amount of CO_2_ injected. Under the condition that the pipeline is shut down to the same liquid holdup of 0.7, if the restart operation is performed at a mass flow rate of 4 kg/s, the required CO_2_ injection mass is 565 t. If performed at 34.7 kg/s, the required mass is 314 t, with the former being 1.8 times that of the latter.

When the injected CO_2_ mass flow rate exceeds 10 kg/s, the liquefaction time under different mass flow rates is short, and the difference is small, all within 5 h. There is little difference in either the restart completion time or CO_2_ consumption, indicating that the mass flow rate becomes a non-sensitive factor after exceeding a certain threshold.

**Figure 25:**
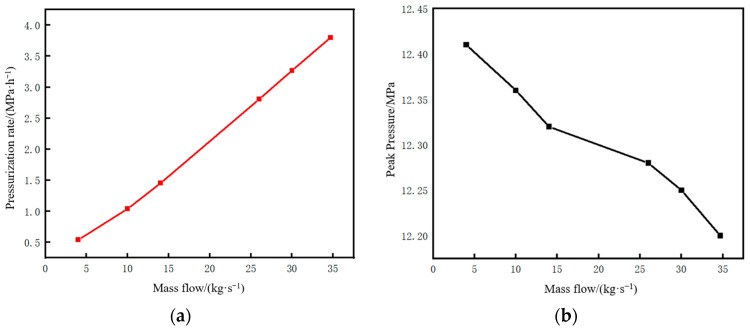
Diagram of pressure rise rate and pressure peak at the initial station during pipeline restart under different injection mass flow rates. (**a**) Pressure rise rate. (**b**) Peak pressure.

The pressure rise rate and pressure peak at the initial station outlet during the pipeline restart process under different injection mass flow rates are shown in Figure 25. It can be seen that the pressure rise rate is approximately proportional to the injected CO_2_ mass flow rate. Under the condition of a liquid holdup of 0.7 at the time of pipeline restart, if the pressure rise rate is controlled to not exceed 1 MPa/h, the injected CO_2_ mass flow rate should not exceed 10 kg/s.

**Figure 26:**
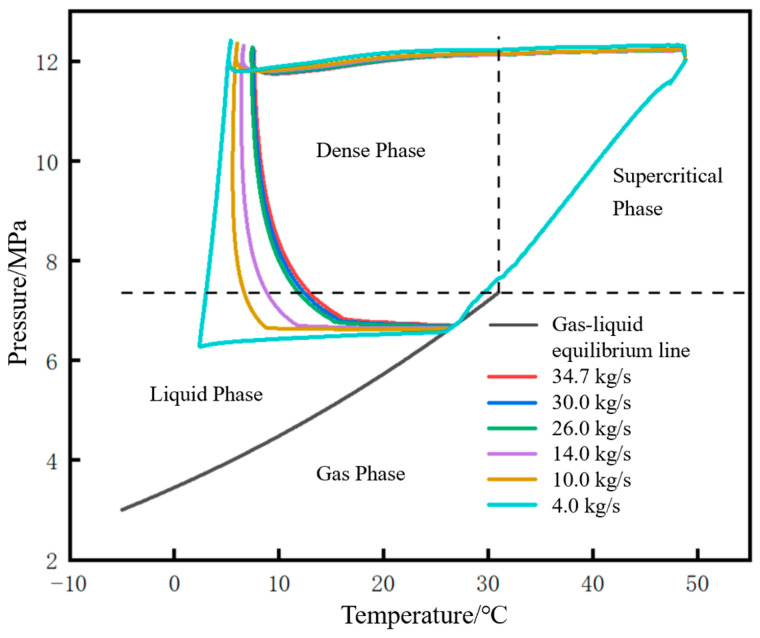
Phase point migration diagram of fluid at pipeline starting point under different injection mass flow rates.

The phase point migration diagrams of the pipeline initial station under different injection mass flow rates are shown in Figure 26. The smaller the mass flow rate, the larger the envelope area. Similar to the phase point migration diagrams at the pipeline initial station under different liquid holdups, the differences among different injection mass flow rates are mainly reflected in the liquid phase region and dense phase region. The smaller the mass flow rate, the smaller the angle between the liquefaction line and the pressurization line. For example, when the injection mass flow rate is 4.0 kg/s, the angle between the two lines is nearly 90°.

### 6.3. Soil Temperature

When the pipeline is shut down under the same fluid pressure and temperature, the time required to reach the same state varies with the season. As shown in Table 8, under three ground temperature conditions (2 °C, 12 °C, and 22 °C), the time required to shut down the pipeline until the internal fluid pressure reaches 6.75 MPa, temperature reaches 27.1 °C, and liquid holdup reaches 0.9 is 5.41 h, 7 h, and 10.48 h, respectively, with the shortest time required under winter conditions.

**Figure 27:**
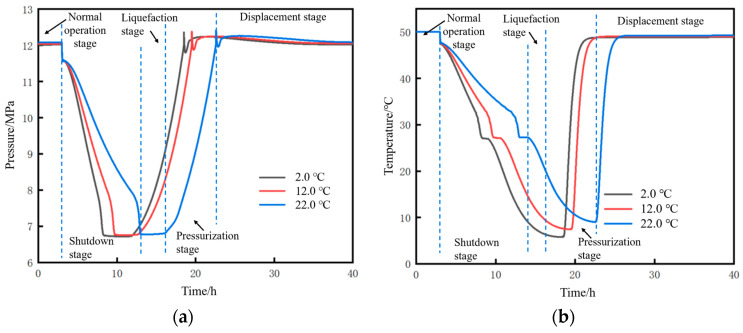
Variation diagram of pressure and temperature at pipeline initial station outlet under different soil temperatures. (**a**) Pressure. (**b**) Temperature.

Based on a liquid holdup of 0.89 at the initial moment of pipeline restart and an injection mass flow rate of 10 kg/s at the initial station, the changes in pressure and temperature parameters at the initial station during the pipeline shutdown and restart processes under different ground temperature conditions were calculated, as shown in Figure 27. For both pressure and temperature, the higher the soil temperature, the longer the time required to return to the state before shutdown.

**Figure 28:**
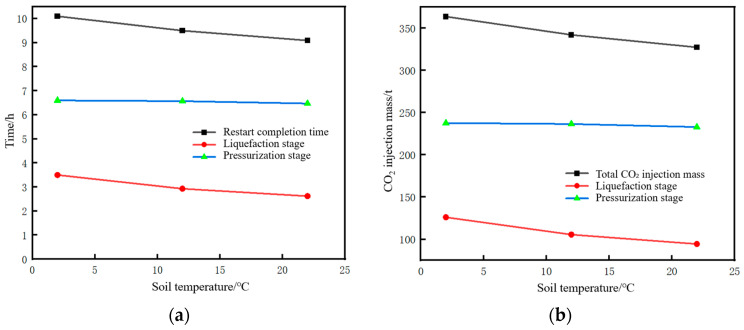
Variation diagram of time parameters and corresponding CO_2_ injection mass during pipeline restart under different soil temperatures. (**a**) Time parameters of the restart process. (**b**) CO_2_ injection mass.

**Figure 29:**
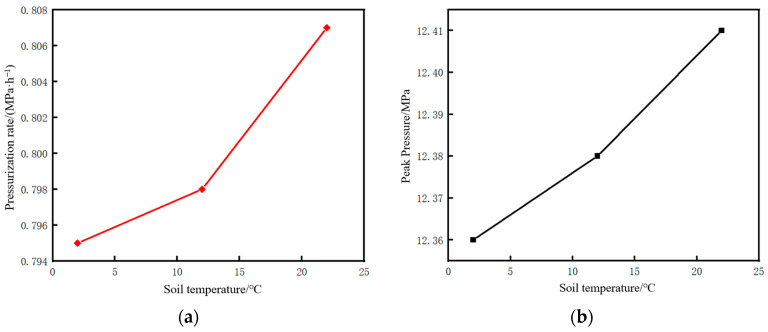
Diagram of pressure rise rate and pressure peak during pipeline restart under different soil temperatures. (**a**) Pressure rise rate. (**b**) Peak pressure.

**Figure 30:**
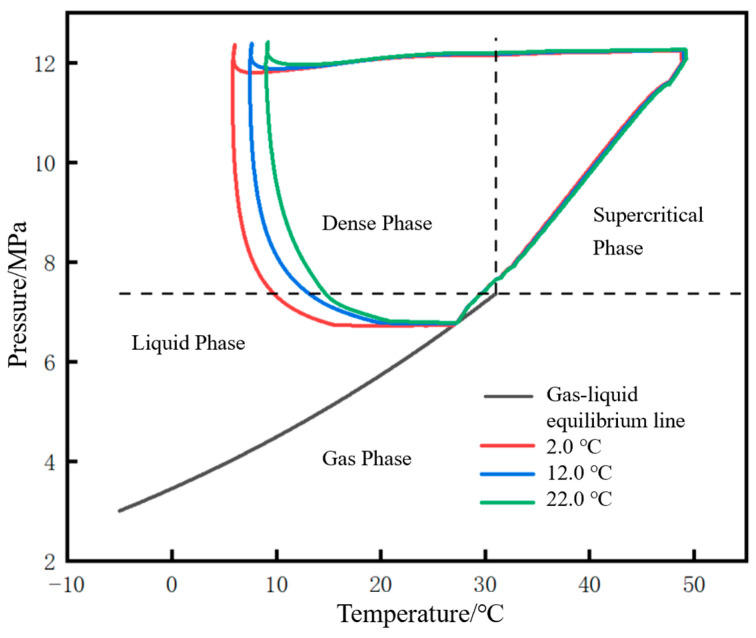
Phase point migration diagram of the pipeline initial station under different soil temperatures.

The time parameters of the pipeline restart process and the corresponding changes in CO_2_ injection mass under different soil temperatures are shown in Figure 28. There is little difference in the restart completion time and CO_2_ injection mass under different soil temperatures, but they are slightly higher under winter conditions. The pressure rise rate and pressure peak during the pipeline restart process under different soil temperatures are shown in Figure 29, and the phase point migration diagrams of the pipeline initial station under different soil temperatures are shown in Figure 30. The differences are also not significant, indicating that ground temperature is a non-sensitive factor.

## 7. Conclusions

(1) When a supercritical CO_2_ pipeline is shut down for an excessively long time, and the fluid inside the pipeline has undergone a phase transition from the supercritical phase to the gas–liquid two-phase state, the pipeline can be restarted by injecting liquid-phase CO_2_ into the initial station. The newly injected liquid-phase CO_2_ exerts a pressure-holding effect on the existing gas–liquid mixture in the pipeline. When the liquefaction pressure is reached, both simulations and experiments show that the phase transition from gas to liquid can be completed. Therefore, it is recommended to install a cooling device downstream of the compressor at the initial station of supercritical CO_2_ pipelines to prepare liquid-phase CO_2_, facilitating restart operations after pipeline shutdown;

(2) The shutdown and restart process of supercritical CO_2_ pipelines can be subdivided into four stages: a shutdown stage, a liquefaction stage, a pressurization stage, and a displacement stage. On the phase diagram, the phase path of the operating point is supercritical phase ⟶ dense phase ⟶ liquid phase ⟶ gas–liquid two-phase ⟶ liquid phase ⟶ dense phase ⟶ supercritical phase. The migration line forms a closed curve that is approximately trapezoidal. The envelope area enclosed by the phase migration lines of the operating points at different positions of the pipeline varies, decreasing sequentially from the upstream to the downstream of the pipeline;

(3) It is suggested to optimize the restart scheme by reducing the restart time, controlling the pressure rise rate, and saving CO_2_ consumption. The liquid holdup of the fluid in the pipeline at the initial moment of restart and the mass flow rate of CO_2_ injected at the initial station during the restart process are sensitive factors affecting the pipeline restart process, while soil temperature is a non-sensitive factor.

(4) A low liquid holdup of the fluid in the pipeline at the initial moment of restart and a low mass flow rate during the restart process will result in a longer liquefaction time and a larger CO_2_ injection mass, i.e., higher costs. A liquid holdup of 0.5 takes 2.5 h longer and requires 90 t more CO_2_ injection than a liquid holdup of 0.9. A mass flow rate of 4 kg/s takes 26 h longer and requires 160 t more CO_2_ injection than a mass flow rate of 10 kg/s;

(5) From a safety perspective, if the pressure rise rate is controlled to not exceed 1 MPa/h, the liquid holdup of the fluid in the pipeline at the initial moment of restart should not be less than 0.7, and the mass flow rate of CO_2_ injected at the initial station during the restart process should not exceed 10 kg/s;

(6) The restart scheme and parameter thresholds proposed in this study can provide a reference for a million-ton-class supercritical CO_2_ pipeline demonstration project, ensuring the safety of pipeline restart. In the future, the influence of impurities, such as methane and nitrogen in CO_2_, on the evolution of CO_2_ pressure, temperature, and phase state during the pipeline restart process will be considered to optimize the restart scheme for multi-component systems. Additionally, combined with on-site monitoring data, a real-time monitoring and dynamic regulation system for the restart process will be developed to realize the intelligent upgrading of pipeline operation and maintenance.

## Figures and Tables

**Table 1:** CO_2_ pipeline foundation parameters.

Parameters	Value
Length/km	52
Design pressure/MPa	13
Pipe diameter	DN250 (Φ 273.1 mm × 8 mm)
Steel grade	L415 M
Inner-wall roughness of pipeline/μm	30
Average ground temperature at the depth of pipe burial in winter/°C	2
Average ground temperature at the depth of the pipeline in summer/°C	22
Annual average ground temperature at the depth of the pipeline/°C	12
Overall heat transfer coefficient/(W·m^−2^·°C^−1^)	1.2
Initial station outlet pressure/MPa	12.0
Terminal station inlet pressure/MPa	>9.0

**Table 2:** Calculated results of gaseous and liquid CO_2_ densities and predicted liquid holdup under different pressures and temperatures.

Pressure/MPa	Temperature/°C	Gaseous Density/(kg·m^−3^)	Liquid Density/(kg·m^−3^)	Predicted Liquid Holdup
7.0	28.1	-	592.1	1.00
6.0	22.1	209.0	685.2	0.80
5.0	14.4	155.6	781.8	0.70
4.0	5.5	115.6	869.7	0.63

**Table 3:** Comparison between calculated and simulated liquid holdup during shutdown after fluid vaporization under different pressures and temperatures.

Pressure/MPa	Temperature/°C	Liquid Holdup (OLGA Simulated Value)	Liquid Holdup (Calculated Value)	Error/%
6.6	26.30	0.94	0.91	−3.19
6.0	21.98	0.81	0.80	−1.23
5.5	18.32	0.77	0.74	−3.90
5.0	14.40	0.72	0.70	−2.78
4.5	10.18	0.67	0.66	−1.49
4.0	5.59	0.64	0.63	−1.56
3.6	2.00	0.62	0.62	0

**Table 4:** Calculation results of main parameters for pipeline restart process.

Phase Transition	Inject Fluid	Injection Temperature /°C	Injection Flow Mass Rate/(kg·s^−1^)	Injected Mass/t	Time Consumed/h	Valve Status at Terminal Station	Stage
Gas–liquid two-phase ⟶ liquid phase ⟶ dense phase (Stage 1)	Liquid phase/dense CO_2_	2.0	10.0	363.6	10.10	Closed	Liquefaction and pressurization stage
Dense phase ⟶ Supercritical phase (Stage 2)	Supercritical CO_2_	50.0	34.7	3184.2	25.49	Open	Displacement stage
Total	-	-	-	3547.8	35.59	-	-

**Table 5:** Parameter changes at different shutdown times.

Time/s	Pressure/MPa	Temperature/°C	Phase State	Liquid Holdup
0	10.41	40.20	Supercritical phase	1
452	7.77	31.05	Supercritical phase ⟶ Dense phase	1
510	7.33	29.60	Dense phase ⟶ Liquid phase	1
588	6.66	26.30	Gas–liquid two-phase	0.9
741	6.02	22.00	Gas–liquid two-phase	0.8
1166	5.00	14.40	Gas–liquid two-phase	0.7

**Table 6:** Values of restart influencing factors.

Influencing Parameters	Liquid Holdup	Initial Station Medium Injection Flow Rate/(kg·s^−1^)	Soil Temperature/°C
Parameter values	0.5	4	2
	0.6	10	12
	0.7	14	22
	0.8	26	-
	0.9	30	-
	-	34.7	-

**Table 7:** Liquid holdup, pressure, and temperature at the initial station corresponding to different shutdown times.

Liquid Holdup	Pressure/MPa	Temperature/°C	Shutdown Time/h
0.9	6.75	27.1	5.41
0.8	6.71	26.8	6.37
0.7	6.64	26.4	8.88
0.6	6.53	25.6	13.75
0.5	6.36	24.5	20.90

**Table 8:** Shutdown time required for the pipeline to reach the same state under different soil temperatures.

Soil Temperature	Pressure/MPa	Temperature/°C	Shutdown Time/h
2.0	6.76	27.1	5.41
12.0	6.76	27.1	7.00
22.0	6.76	27.1	10.48

## Data Availability

The original contributions presented in this study are included in the article. Further inquiries can be directed to the corresponding author.
